# Respiratory syncytial, parainfluenza and influenza virus infection in young children with acute lower respiratory infection in rural Gambia

**DOI:** 10.1038/s41598-019-54059-4

**Published:** 2019-11-29

**Authors:** Grant A. Mackenzie, Aminata Vilane, Rasheed Salaudeen, Lenny Hogerwerf, Sharon van den Brink, Lisa A. Wijsman, Pieter Overduin, Thierry K. S. Janssens, Thushan I. de Silva, Marianne A. B. van der Sande, Beate Kampmann, Adam Meijer

**Affiliations:** 1 Medical Research Council Unit The Gambia at London School of Hygiene and Tropical Medicine, Fajara, The Gambia; 2 Murdoch Children’s Research Institute, Melbourne, Australia; 3 Department of Disease Control, Faculty of Infectious and Tropical Diseases, London School of Hygiene & Tropical Medicine, London, UK; 4 Institut de Recherche en Santé, de Surveillance Epidémiologique et de Formations, Dakar, Senegal; 5 National Institute for Public Health and the Environment, Centre for Infectious Diseases, Epidemiology and Surveillance, Bilthoven, The Netherlands; 6 National Institute for Public Health and the Environment, Centre for Infectious Diseases Research, Diagnostics and laboratory Surveillance, Bilthoven, The Netherlands; 7 Centre of International Child Health, Section of Paediatrics, Department of Medicine, Imperial College London, St Mary’s Campus, London, UK; 8 Department of Public Health, The Institute of Tropical Medicine, Antwerp, Belgium; 9 Julius Center for Health Sciences and Primary Care, University Medical Center Utrecht, Utrecht, Netherlands; 10 The Vaccine Centre, Department of Clinical Research, Faculty of Infectious and Tropical Diseases London School of Hygiene and Tropical Medicine, London, UK

**Keywords:** Infectious diseases, Viral infection

## Abstract

Respiratory viral infections contribute significantly to morbidity and mortality worldwide, but representative data from sub-Saharan Africa are needed to inform vaccination strategies. We conducted population-based surveillance in rural Gambia using standardized criteria to identify and investigate children with acute lower respiratory infection (ALRI). Naso- and oropharyngeal swabs were collected. Each month from February through December 2015, specimens from 50 children aged 2–23 months were randomly selected to test for respiratory syncytial (RSV), parainfluenza (PIV) and influenza viruses. The expected number of viral-associated ALRI cases in the population was estimated using statistical simulation that accounted for the sampling design. RSV G and F proteins and influenza hemagglutinin genes were sequenced. 2385 children with ALRI were enrolled, 519 were randomly selected for viral testing. One or more viruses were detected in 303/519 children (58.4%). RSV-A was detected in 237 and RSV-B in seven. The expected incidence of ALRI associated with RSV, PIV or influenza was 140 cases (95% CI, 131–149) per 1000 person-years; RSV incidence was 112 cases (95% CI, 102–122) per 1000 person-years. Multiple strains of RSV and influenza circulated during the year. RSV circulated throughout most of the year and was associated with eight times the number of ALRI cases compared to PIV or IV. Gambian RSV viruses were closely related to viruses detected in other continents. An effective RSV vaccination strategy could have a major impact on the burden of ALRI in this setting.

## Introduction

The introduction of conjugate vaccines against *Haemophilus influenzae* type b and *Streptococcus pneumoniae* in many countries has contributed to reductions in pneumonia deaths over the last two decades^1,2^. The introduction of pneumococcal conjugate vaccine in The Gambia was associated with a 61% reduction in severe hypoxic pneumonia^3^. However, the incidence of all-cause acute lower respiratory infection (ALRI) remains high^3^ with an estimated 921,000 deaths due to ALRI in children <5 years of age in 2015, 490 000 of which occurred in sub-Saharan Africa^1^.

In order to address the ongoing burden of virus-associated ALRI and to develop evidence-based vaccination strategies, additional data are needed from low-income settings. Recent case-control studies from low-income countries have established an etiologic association between ALRI and respiratory syncytial (RSV), parainfluenza (PIV), influenza (IV) and human metapneumo viruses^4,5^. However, these studies could not calculate disease incidence. A recent systematic review estimated the global number of cases of RSV-associated ALRI in 2015 at 33.1 million, 3.2 million hospital admissions and 59 600 deaths with 45% of cases occurring before 6 months of age^6^. Incidence varied by location, with only three (two unpublished) African studies reporting disease incidence^7^, with none in West Africa. Thus, additional African data are urgently needed as several RSV vaccine candidates are in development with consideration of maternal, neonatal and infant vaccination strategies^8^.

Here we present the incidence of ALRI associated with RSV, PIV or IV in young children in The Gambia, including the clinical features and temporal circulation. We also evaluate specimen collection and storage methodologies and analyse antigenic sites that may impact on vaccination strategies.

## Methods

### Study setting

The Gambia is a small West African country with a population of approximately 2.1 million. The Basse Health and Demographic Surveillance Systems (BHDSS) in the rural east of the country had an estimated population of 179 932 in 2015 with 12,318 aged 2–23 months. The Basse Health Centre is a primary and secondary care facility in Upper River Region of The Gambia, providing referral services to five peripheral health facilities in the BHDSS. HIV prevalence in antenatal attendees was 1.6% in 2014^9^. Transmission of *Plasmodium falciparum* is associated with a rainy season from June until October. 7-valent pneumococcal conjugate vaccine (PCV7) was introduced in 2009 and replaced by PCV13 in 2011.

### Study design and procedures

This study was nested within a population-based surveillance study for suspected pneumonia, septicemia and meningitis, designed to measure the impact of PCV introduction. Surveillance methodology^10^ and results^3,11^ have been previously published. Surveillance commenced in 2008 and included all BHDSS residents. We added the collection of nasopharyngeal (NP) and oropharyngeal (OP) swabs from all surveillance patients from February 2015 onwards with the aim to measure pneumococcal carriage. The study presented here used data and stored specimens collected between February 10 and December 31, 2015.

Surveillance nurses screened all outpatients and inpatients at all health facilities in the BHDSS, 24 hours a day, 7 days per week, using standardized criteria for referral to clinicians (Supplementary Table S1). Clinicians applied standardized criteria to make a surveillance diagnosis (Supplementary Table S2) and requested blood culture, chest radiography, NP and OP specimens according to a standardized protocol (Supplementary Table S3). Flocked nylon swabs (Copan, Murietta, CA, USA) were used to collect separate NP and OP specimens^12^.

Specimens were placed in skimmed-milk-tryptone-glucose-glycerol (STGG) and transported within one hour to the laboratory in Basse and stored at −70 °C.

We defined ALRI as cough or difficulty breathing for 14 days or less and one or more of the following: raised respiratory rate for age, lower chest wall indrawing, nasal flaring, grunting, oxygen saturation <92%, altered consciousness, prostration, seizures, dull chest percussion note, coarse crackles, or bronchial breathing. Children were eligible if aged 2–23 months, admitted to hospital, and excluded if the surveillance diagnosis was septicemia or meningitis alone without suspected pneumonia. Children with ALRI and proven invasive bacterial disease were included.

### Specimen processing

#### Validation of STGG media for molecular viral diagnosis

To validate virus detection in STGG, we simulated clinical specimens using two different media, STGG or gelatin, lactalbumin hydrolysate, yeast extract in Hank’s medium (GLY) virus transport medium (gold standard) spiked with virus. First, serial 10-fold dilutions of specimens containing IV A(H3N2), A(H1N1)pdm09, B-Victoria lineage, B-Yamagata lineage, RSV-A and RSV-B were prepared in GLY. Duplicate specimens from each dilution were prepared by mixing 2 μl of diluted specimen with 200 μl STGG or GLY. All specimens were also spiked with control equine arteritis virus (EAV). RNA was extracted manually (Total RNA kit, Roche). The duplicate specimens were subjected to real-time RT-PCR for IV, RSV and EAV.

In order to validate the molecular detection method in field samples, we first randomly selected approximately 50 patients in both August and September 2015 who met our definition of ALRI. Stored aliquots of NP and OP specimens from these patients were used in validation experiments at the Centre for Infectious Diseases Research, Diagnostics and *laboratory* Surveillance at the National Institute for Public Health and the Environment (RIVM), the Netherlands.

#### Analysis of randomly selected field specimens

Following the demonstration of successful detection of viral nucleic acid and the validation and concordance of viral detection in NP and OP specimens, we went on to randomly selected approximately 50 patients per month from February through July and October through December 2015. Thus, the overall analysis included specimens from 11 months (February through December 2015).

Extraction of nucleic acid from the clinical specimens and control material in STGG was performed on a MagNA Pure 96 Instrument (Roche) using the MagNA Pure 96 DNA and Viral NA Small Volume Kit (Roche). Specimens and controls were spiked with EAV as an internal control. One-step real-time reverse transcription (RT)-PCR using TaqMan Fast Virus 1-step Master Mix (Applied Biosystems Life Technologies) was done for the detection and typing of IV, RSV, PIV and EAV. We determined IV subtyping and lineage using the same approach. The Primers and probes used and the PCR protocol are listed in Supplementary Table S4. For each RT-PCR positive control (strains indicated in Supplementary Table S5), two negative controls consisting of STGG and GLY were included.

#### Sequencing of RSV and influenza virus

We sequenced the length of the G-protein gene encoding part of the transmembrane portion and the whole external part of the G-protein and part of the F-protein gene covering antigenic sites Ø, II and VIII from selected RSV positive specimens using protocols adapted from Agoti *et al*.^13^. and Xia *et al*.^14^. The full length hemagglutinin gene was sequenced from selected IV positive specimens. All primer sequences and protocols used can be found in Supplementary Tables S6–S9. Viruses were selected based on viral load [PCR Cycle threshold (Ct) value <30] and distribution across the months of specimen collection.

### Analysis

We restricted the analysis of clinical characteristics to children resident in the BHDSS area. Categorical data were used to calculate proportions and comparisons used the χ^2^ or Fisher’s exact test. Mean values for continuous data were compared using t-tests. Tests of association were two-sided with statistical significance set at *p* < 0.05.

The expected value and 95% confidence limits for the total number of cases among all children resident in the BHDSS and presenting with ALRI between 1 February and 31 December 2015 were derived by simulation taking into account the monthly sampling of patients resident in the study area (Supplementary Material p 19). We simulated the monthly proportions of children with viral-associated ALRI, assuming a binomial distribution with the observed proportion of positive samples from the number of patients sampled each month. We then randomly sampled from an independent binomial distribution with these proportions, from the whole population of ALRI cases to estimate the number of positive cases each month at the population level. This was repeated 1,000,000 times for each month and the expected number of positive cases and their 95% confidence limits were estimated as the mean and 2.5% and 97.5% percentiles of the simulated values. We used these expected values and the mid-point population (12,318 × [11/12]) to calculate the expected incidence of ALRI associated with each of the target viruses with 95% confidence limits. Simulations used R v3.43 software.

In order to study the viruses circulating in the region, isolates detected in children resident within and those resident outside the BHDSS were eligible for selection for genome sequencing. We used the sequences of Gambian RSV and IV from this study, selected sequences extracted from GenBank for RSV and GISAID for IV, and selected sequences of RSV and IV from Dutch surveillance for influenza-like illness and other acute respiratory infections to infer phylogenetic trees of the genetic relatedness of viral genes encoding the G-protein of RSV-A and RSV-B and the hemagglutinin gene of IV (Supplementary Methods p. 21–22, phylogenetic analysis and Supplementary Table 14). The RSV G-protein and IV hemagglutinin amino acid sequences were analysed for substitutions compared to parental strains for RSV or vaccine strains for IV. RSV F-protein gene sequences were analysed for antigenic site composition in the context of RSV-A strain A2 and RSV-B strain B1 that are frequently used in vaccine development^15^. In addition, potential N-glycosylation sites were identified for RSV F- and G-proteins and IV hemagglutinin and potential O-glycosylation sites for RSV G-protein.

### Ethical considerations

The Gambia Government/MRC Institutional Ethics Committee approved the study which was conducted in accordance with the relevant guidelines and regulations. Parents or guardians of all children gave written informed consent.

## Results

### Participant enrolment and characteristics

During the observation period the surveillance system enrolled 2518 children aged 2–23 months, 2385 met criteria for ALRI and 532 specimens were randomly selected for viral testing (519 BHDSS residents).

### Validation of STGG media for virus detection

Ct values for IV A and B controls generated from RNA extracted from STGG were consistently slightly higher (average 1.2 and 1.0 higher Ct respectively) than those generated from RNA extracted from the gold standard GLY, and similar was observed for RSV A and B controls (average 1.2 and 1.4 higher Ct respectively). The fraction positive for RSV and IV among the four replicates per dilution started to drop one 10-fold dilution step earlier with RNA extracted from STGG compared to GLY. Taken together these findings suggest a minor but clear inhibiting effect of STGG on RNA extraction and/or RT-PCR efficiency. We conclude that detection of IV and RSV in STGG is up to 10 times less sensitive than in GLY. Despite the reduced sensitivity of viral detection in STGG, the collection of clinical specimens in the acute phase of disease when viral load is high still provides good clinical performance for the detection of IV, RSV and PIV.

### Virus detection in clinical specimens

**Figure 1:**
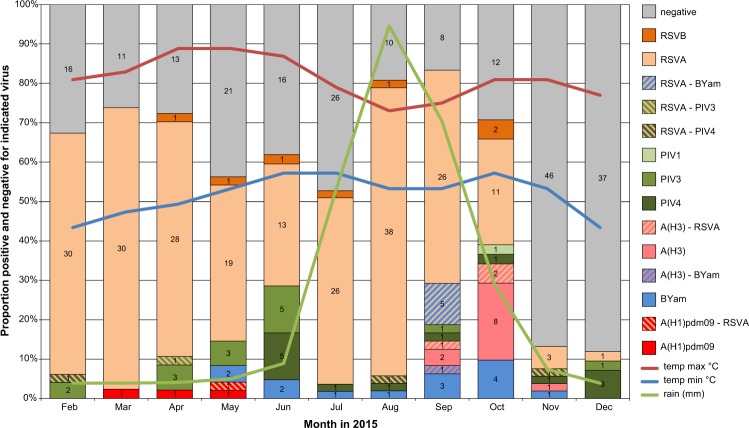
Proportion (bars) and number of positive cases (numbers in bars) per pathogen per month. Cases positive for two viruses are shown as one case. Indicated are also the minimum (lowest 19 °C in January) and maximum (highest 43 °C in April and May) temperatures in °C and rainfall in mm (lowest 0 mm, highest 275.5 mm in August) per month at weather station Tambacounda, Senegal, most close to Upper River Region, The Gambia (https://www.worldweatheronline.com/basse-weather-averages/upper-river/gm.aspx).

STGG specimens were tested from a total of 532 patients (13 not resident in the BHDSS) with an average of 48 (range 42–58) per month from February through December 2015. One or more viruses were detected in 303/519 patients (58.3%) with 14 cases (5%) of co-infection (Fig. 1). RSV-A was detected in 237/519 (45.9%) patients, and constituted the predominant virus in the dry season months February through June as well as during the rainy season July through October (Fig. 1). RSV-B was uncommon, detected in only seven patients. PIV-1 was detected in only one patient, while PIV-3 was detected in 16 (one with RSV-A co-infection) mainly in the first half of the year and PIV-4 was detected in 16 (three as a co-infection with RSV-A) primarily in the second half of the year (Fig. 1). A small number of influenza A(H1N1)pdm09 cases (n = 5) were detected early in the year with A(H3N2) appearing later in the year (n = 15). B/Yamagata infections (n = 19) began when A(H1N1)pdm09 infections started to drop and peaked at the same time as A(H3N2) viruses in the rainy season months of September and October (Fig. 1). In general, OP swabs had higher Ct values than NP swabs indicative of a difference in viral load (Table 1). Detection of RSV was greater in NP versus OP specimens.

**Table 1:** Concordance of results between nasopharyngeal (NP) and oropharyngeal (OP) specimens.

Pathogen; N	Concordant			Discordant			
	n	NP +ve	OP +ve	NP +ve, OP −ve		NP −ve, OP +ve	
		(Ct median; IQR)	(Ct median; IQR)	n	(Ct median; IQR)	n	(Ct median; IQR)
RSV-A; 237*	206	(26.4; 24.2–28.8)	(30.7; 28.5–33.2)	25	(32.2; 30.5–35.8)	6	(29.2; 24.7–32.7)
RSV-B; 7	5	(27.8; 26.8–29.4)	(30.5; 30.4–32.9)	1	(33.2; na)	1	(40.0; na)
Influenza A; 19	17	(29.6; 26.6–31.2)	(33.0; 30.1–33.7)	1	(32.8; na)	1	(33.1; na)
Influenza B; 20	17	(27.4; 23.4–30.8)	(30.9; 28.5–32.6)	1	(36.1; na)	2	(33.0; na)
PIV-1; 1	0			1	(31.9; na)	0	
PIV-3; 16	6	(28.2; 26.8–28.5)	(36.1; 32.2–37.6)	10	(27.6; 25.3–29.6)	0	
PIV-4; 16	9	(30.3; 29.9–31.7)	(32.6; 31.8–36.0)	6	(31.4; 30.5–33.0)	1	(37.9; na)

^*^One patient positive for RSV-A not included in this analysis because no oropharyngeal swab was available.

Ct = real-time RT-PCR cycle threshold value; IQR = interquartile range; +ve = positive; −ve = negative; na = not applicable.

### Clinical features of patients

The demographic and clinical features of the selected patients with ALRI (n = 519) were similar to those who were not selected (n = 1866) [Supplementary Table S10]. Table 2 shows the clinical characteristics of the 519 children according to four categories of ALRI. Wheeze was more common among children with detectable virus compared to those without (*p* = 0.005). The prevalence of viral detection was higher in those aged 2–11 months (61.1%, 196/321) than 12–23 months (54.0%, 107/198), although the difference was not significant (*p* = 0.12). However, RSV was more commonly detected in those aged 2–11 months (51.4%, 165/321) than those aged 12–23 months (36.4%, 72/198) [*p* = 0.0008].

**Table 2:** Clinical characteristics of a representative sample of 519 children aged 2–23 months with different categories of acute lower respiratory infection in 2015 and tested for RSV, PIV and IV, in rural Gambia.

	No virus detected & no radiologic pneumonia or bacteremia (N = 185)	≥1 virus detected & no radiologic pneumonia or bacteremia (N = 274)	Radiologic consolidation with or without viral detection (N = 52)	All selected for viral testing (N = 519)
Age 2–11 months	109 (58.9%)	180 (65.6%)	27 (52%)	321 (61.9%)
Age 12–23 months	76 (41.1%)	94 (34.3%)	25 (48%)	198 (38.2%)
Mean age in months (SD)	11.3 (5.9)	10.3 (6.3)	11.8 (6.5)	10.8 (6.2)
Female	82 (44.3%)	123 (44.9%)	29 (56%)	236 (45.5%)
Mean respiratory rate per minute (SD)	53 (10)	56 (10)	59 (14)	55 (11)
Wheeze	42 (22.7%)	96 (35.0%)	9 (17%)	150 (28.9%)
Lower chest wall indrawing	74 (40%)	159 (58.0%)	29 (56%)	264 (50.9%)
Mean O_2_ saturation (SD)	97.4 (7.0)	96.7 (3.2)	94.5 (5.9)	96.8 (5.2)
Treated as inpatient	84 (45.4%)	159 (58.0%)	42 (81%)	291 (56.1%)
Inpatient death	1 (<0.1%)	0 (0.0%)	1 (2%)	3 (0.6%)

Note: Eight patients had bacteremia and no virus detected. SD, standard deviation.

### Incidence of acute lower respiratory infection

There were 303 cases of ALRI associated with at least one of the target viruses observed among the 519 selected resident patients (Table 3 and Supplementary Table S11). Simulation of the expected number of cases in all patients with ALRI during the observation period took into account the random monthly sampling and found an expected 1543 cases (Table 3 and Supplementary Tables S11–S13). The expected incidence of ALRI associated with RSV, PIV or IV was 140 cases per 1000 population per year (Table 3); that is, we estimated that 14% of 2–23 month old children in the population presented with virus-associated ALRI in 2015. The incidence of RSV-A associated ALRI was 112 per 1000 population per year; we therefore estimated that 11% of the 2–23 month old population presented with ALRI associated with RSV-A. Incidence of viral-associated ALRI was substantially greater in the first compared to the second year of life (Table 3).

**Table 3:** Incidence of virus-associated acute lower respiratory infections in children aged 2–23 months from February to December 2015 in the Basse HDSS, rural Gambia; (mid-point population at risk: 2–11 mo = 5038, 12–23 mo = 6254).

Age 2–11 mo	Observed number of cases	Expected number of cases (95% CI)	Expected incidence per 1000 population per year (95% CI)
Any virus	196	991 (913, 1066)	197 (181, 212)
RSV-A	165	847 (765, 927)	168 (152, 184)
RSV-B	3	14 (0, 35)	3 (0, 7)
Influenza-A	9	51 (21, 85)	10 (4, 17)
Influenza-B	6	31 (8, 59)	6 (2, 12)
PIV-1	1	6 (0, 20)	1 (0, 4)
PIV-3	10	47 (19, 81)	9 (4, 16)
PIV-4	10	35 (12, 62)	7 (2, 12)
**Age 12–23 mo**			
Any virus	107	561 (494, 626)	90 (79, 100)
RSV-A	72	389 (321, 457)	62 (51, 73)
RSV-B	4	22 (3, 47)	4 (0.5, 8)
Influenza-A	10	54 (23, 91)	9 (4, 15)
Influenza-B	14	74 (37, 115)	12 (6, 18)
PIV-1	0		
PIV-3	6	28 (7, 55)	4 (1, 9)
PIV-4	6	33 (8, 64)	5 (1, 10)
**Age 2–23 mo**			
Any virus	303	1543 (1439, 1644)	140 (131, 149)
RSV-A	237	1232 (1124, 1339)	112 (102, 122)
RSV-B	7	34 (10, 65)	3 (1, 6)
Influenza-A	19	97 (55, 144)	9 (5, 13)
Influenza-B	20	104 (60, 154)	9 (5, 14)
PIV-1	1	5 (0, 18)	0.5 (0, 2)
PIV-3	16	77 (40, 120)	7 (4, 11)
PIV-4	16	67 (33, 107)	6 (3, 10)

*Note:* Values for the expected and 95% confidence limits for the number of cases in the population took into account the monthly sampling scheme, being generated by simulation using the number of observed cases each month, the number of patients tested each month and the proportion of all patients tested each month (see Methods and Supplementary Tables 11–13 and Supplementary Material p 19). Expected numbers of cases by simulation in age strata may not sum to the expected number of cases overall.

### Viral genetic sequencing

**Figure 2:**
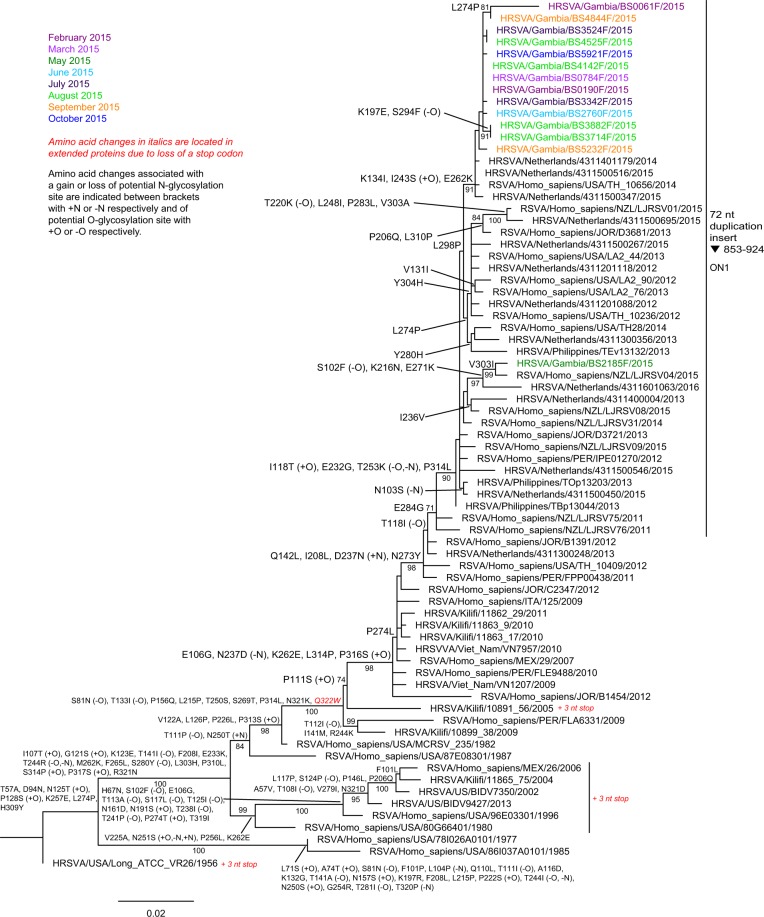
Phylogenetic tree for RSV-A, based on partial sequences of the G-protein gene. Protein sequence ranges from amino acid position 54 up to stop codon. +N and −N indicate gain and loss of N-glycosylation sites and +O and −O indicate gain and loss of O-glycosylation sites. Country codes in the virus names: ITA = Italy; JOR = Jordan; Kilifi = Kenya; MEX = Mexico; NZL = New Zealand; PER = Peru; US or USA = United States of America.

Phylogenetic trees using the RSV G-protein gene and the IV hemagglutinin gene sequencing from 19 RSV and 16 IV strains selected throughout the study period, are found in Fig. 2 and Supplementary Figs S1–S5. All Gambian RSV-A viruses clustered in the ON1 clade and all but one of the sequenced RSV-A viruses clustered with 2014 and 2015 viruses from The Netherlands and the USA in a subgroup of clade ON1 characterised by specific amino acid substitutions. Further diversification was seen characterised by particular common amino acid substitutions. One Gambian RSV-A clustered with 2015 and 2016 viruses from New Zealand and The Netherlands in another subgroup of clade ON1 characterised by specific amino acid substitutions. All five sequenced RSV-B clustered in a subgroup of clade BA characterised by specific amino acid substitutions with 2013–2016 RSV-B viruses from other continents. One Gambian RSV-B virus lost two stop codons resulting in a longer G-protein. Several of the amino acid substitutions of the clusters containing the Gambian RSV-A or RSV-B resulted in the gain or loss of potential O-glycosylation sites in the G-protein. Less complete G-protein gene sequences from Kenya in 2014–2015 did not cluster with Gambian RSV-A sequences (Supplementary Fig. S1). For RSV-B, a small proportion of 2015 and all 2016 Kenyan RSV-B, clustered with Gambian RSV-B in a larger subgroup of clade BA (Supplementary Fig. S1). Full details of the G-protein sequence analysis can be found in the Supplementary Material p. 31. Gambian RSV F-protein sequences showed that key antigenic sites were highly conserved, although some amino acid differences were observed between RSV-A and RSV-B (Supplementary Fig. S6). RSV-B had one N-glycosylation site less than RSV-A at amino acid 126 due to T128L difference. Circulating IV genotypes were similar to the rest of Africa and The Netherlands in 2015, with similar amino acid changes compared to the recommended vaccine strains (Supplementary Material p. 32).

## Discussion

Our analyses from population-based surveillance in 2015 in rural Gambia provide reliable data on the incidence of ALRI associated with RSV, PIV and IV, its clinical characteristics and a detailed description of the circulation of RSV, PIV and IV throughout the year with genotypic descriptions of the prevalent RSV and IV. RSV-A was the dominant virus detected with a very high incidence of associated ALRI.

Unlike the seasonal circulation of RSV that is generally observed in temperate^16–18^ and tropical climates^19–21^, RSV-A circulated almost throughout the year, similar to observations made in Senegal in 2012 and 2013 but not 2014^22^. PIVs, and IVs in particular, circulated for shorter periods of time. The predominance of one particular RSV type in one season, namely RSV-A in 2015, was similar to that observed in Mali (RSV-B in 2013)^23^, Senegal (RSV-B in 2012, RSV-A in 2013, RSV-B in 2014)^22^, Ghana (RSV-B in 2006 and 2013, RSV-A in 2014)^21^, and the United States (RSV- in 2015)^16^.

We used a monthly sampling design to estimate the expected number of cases during the observation period and estimated that 14% of children aged 2–23 months in the population presented with ALRI associated with RSV, PIV or IV and that 11% presented with ALRI associated with RSV-A. The incidence of any target virus and RSV-A associated ALRI within this demographically well-defined population was 140 and 112 cases per 1000 population per year respectively. The three target viruses were highly prevalent, one or more being detected in 58% of children with ALRI and RSV-A detected in 46% of children. The number of ALRI cases associated with IV and PIV respectively, was only 14% and 13% of the number associated with RSV.

Our finding of 46% prevalence of RSV-A in children with ALRI is higher than in most other studies, suggesting that we observed a particularly intense RSV epidemic in 2015. In coastal Kenya the prevalence of RSV in children aged <5 years hospitalized with pneumonia from 2002–2007 was 15% (20% among infants) and 27% during epidemics (32% among infants)^7^. RSV prevalence was 36% in active case detection of clinical pneumonia in children aged <2 years in Mirzapur, Bangladesh in 1993–1996^24^. During the conduct of the PERCH study of the etiology of childhood pneumonia in 2012/13 in Basse, the prevalence of RSV in children hospitalized with ALRI was 19.7%^25^.

The estimated incidence of RSV-A associated ALRI in our study is particularly high. We estimated an incidence of 112 cases per 1000 population per year (168 per 1000 population per year <1 year of age) while other African studies using passive case detection have reported incidences of 11^7^ and 30^26^ per 1000 person-years in children <1 year of age. Even in studies using active case detection the incidence of RSV disease has generally been similar to our study which used passive case detection. Reanalysis of active case detection data from a number of locations for a systematic review of RSV-associated ALRI found incidence in the first year of life of 38 in India, 88 in Indonesia, 105 in Kenya, 149 in Bangladesh, 148 in Guatemala, 116 in Nigeria and 331 in South Africa^6^. Our estimated incidence of RSV disease in the first year of life was greater than in all these studies, which had used active case detection, apart from the South African study, indicating a very high burden in our setting. A recent study from Mali using active surveillance in infants less than 6 months of age reported a very high incidence of RSV detection at 537 per 1000 person-years^23^. We estimated that in a 12 month period, 16.8% of all infants in the population presented to health facilities with RSV-associated ALRI.

Similar to previous evaluations of the use of swabs collected in STGG for the detection of respiratory viruses, we showed that this is feasible^27,28^, which is important for other investigators wishing to generate results from specimens stored in STGG. However, we also found some loss of sensitivity compared to the gold standard virus transport medium. As the children were sampled at the acute phase of disease when virus shedding is high, this was however not a significant problem for our study. Our data confirm that collection of both OP and NP specimens is associated with greater sensitivity^29,30^. Use of NP swabs alone would result in slight under-reporting of all pathogens. We found greater detection of RSV in NP compared to OP specimens (Table 1), whereas others have described comparable detection in OP and NP specimens^29,30^.

Phylogenetic sequence analysis of RSV-A and RSV-B G-protein genes showed that the Gambian viruses belonged to the recent globally spread clades ON1 and BA respectively and clustered with viruses from other continents, similar to the analysis of Kenyan RSV isolates, and suggesting intra- and inter-continental circulation of RSV strains^31–34^. Separate analysis using less complete Kenyan G-protein gene sequences indicated no strong link between RSV-A viruses circulating in West and East Africa in 2015 (Supplementary Fig. S1). Only for Gambian RSV-B did we notice some association with Kenyan RSV-B from 2015 and 2016 (Supplementary Fig. S1). The subgroups of ON1 and BA to which the Gambian RSV belonged had specific patterns of gain and loss of potential O-glycosylation sites that might affect antigenicity and therefore allow repeat infection^35,36^. Many RSV vaccines under development target site Ø of the pre-fusion form of the F-protein^37^. Recently, a new potent neutralizing antigenic epitope VIII has been identified in the pre-fusion protein F^38^. Similar to previous findings, site Ø and site VIII of Gambian RSV viruses were conserved among RSV-A and RSV-B but of different composition between both types^39^, an aspect relevant to the development of vaccines targeting pre-fusion F-protein. As site Ø of Gambian RSV has the wildtype profile, vaccines under development are expected to accurately target Gambian RSV. Nevertheless, observed differences in the G-protein and F-protein nucleotides and amino acids (Supplementary Material p. 31) indicate that prospective monitoring of F-protein antigenic sites would be required to monitor for the emergence of escape variants following the introduction of RSV immunization in The Gambia.

Currently, no influenza vaccines are used in The Gambia. Given the IV profiles and circulation dynamics, similar to those seen in Senegal (www.who.int/flunet) where an early and late peak in the same year are generally observed every second year, early use of the previous year Northern Hemisphere (NH) recommended vaccine composition and a switch to that for the Southern Hemisphere (SH) for vaccination later in the season could be considered. However, it may be that recommendations for the SH vaccine would be too late for a vaccination campaign in late January/early February to cover the potential March-April circulation of IV. Our results showed that the global recommendations for the A(H1N1)pdm09 vaccine strain were appropriate for The Gambia but there was a mismatch for A(H3N2). NH recommendations for B/Yamagata were mismatched and the recommended SH vaccine was appropriate (see Supplementary Material p. 32).

Our study had a number of limitations. Due to the inability to detect all cases in the population our estimates of incidence are minimal estimates of the true incidence. The observation period of 11 months was not able to capture the known year-to-year variability in the prevalence and seasonality of different respiratory viruses. We excluded those aged <2 months, and although the peak age for RSV disease is between 3–11 months^6^, infants less than 2 months of age are at risk of severe disease. We tested for the three respiratory viruses expected to be most prevalent and did not include viruses such as human metapneumovirus. Inclusion of a larger number of viruses could have closed part of the diagnostic gap, especially for the months of November and December. The testing of only 22% of children with ALRI for viruses was largely overcome by a scheme of random monthly sampling and simulation of the expected number of unobserved cases. This approach provided a valid point estimate of incidence but wider confidence intervals. The ability to detect RSV and IV from swabs stored in STGG compared to the gold standard virus transport media can be generalised to PIV as all are enveloped viruses.

In conclusion, the very high incidence of RSV among young children with ALRI confirms the significant burden of RSV disease, being eight times that of IV or PIV, and the potential for RSV transmission causing disease for up to 9 months of the year. Ongoing surveillance will improve our understanding of seasonal variation and the clinical impact of viral-associated ALRI. Furthermore, we have demonstrated that surveillance for viral pathogens is possible using STGG media, and so may easily be combined with studies of bacterial colonization. With several RSV vaccines in the late stages of development, our insights into intra- and inter-continental transmission of RSV, continental and local genetic variability and evolution, transmission and disease burden, will be critical to the design of vaccines and their evaluation in the future. Similarly, local baseline data are important to support deliberations about future national vaccine introductions. The novel methods and data generated by our integrated clinical, epidemiological and microbiological surveillance can guide the introduction of suitable RSV vaccines, by providing an essential baseline to assess their potential impact and guide future vaccination strategies, including the vaccination of women in pregnancy.

## Supplementary information

Supplementary Information

## Data Availability

The data generated during this study are available from the corresponding author on reasonable request and with approval from the Gambia Government/MRC Joint Ethics Committee.
