# Supplementary material for: Respiratory syncytial, parainfluenza and influenza virus infection in young children with acute lower respiratory infection in rural Gambia
Source: Sci Rep. 2019 Nov 29;9:17965. doi: 10.1038/s41598-019-54059-4 (PMC6884537; doi:10.1038/s41598-019-54059-4)
Supplement: Supplementary file 1 — Supplementary Information [file 41598_2019_54059_MOESM1_ESM.pdf]

Supplementary Information

Respiratory syncytial, parainfluenza and influenza virus infection in young children with acute lower respiratory infection in rural Gambia

*Grant A Mackenzie, Aminata Vilane, Rasheed Salaudeen, Lenny Hogerwerf, Sharon van den Brink, Lisa A Wijsman, Pieter Overduin, Thierry KS Janssens, Thushan I de Silva, Marianne AB van der Sande, Beate Kampmann**, Adam Meijer**. **Beate Kampmann and Adam Meijer contributed equally to this work.

Table S1: Screening criteria for referral of out- and in-patients for clinician assessment (if one or more criteria are present for 14 days or less)

Patients aged 2 to 23 months

- History of cough or difficulty breathing, plus raised respiratory rate for age
- Lower chest wall indrawing, nasal flaring, or grunting
- Oxygen saturation less than 92%
- History of convulsion
- Impaired consciousness*
- Bulging fontanelle
- Stiff neck
- Axillary temperature at least 38°C, or less than 36°C, in a patient admitted or being admitted
- Prostration†
- Weight below -3 z score for age
- Local musculoskeletal swelling or tenderness
- **Irrespective of age or residential location, any child with possible meningitis**

*Impaired consciousness is defined as V, P, or U on the AVPU score, where A is if the patient is alert, V if responsive to verbal stimulus, P if responsive to pain stimulus, and U if unresponsive. †Prostration is defined as an inability to drink or breast feed, or to remain in a seated position in a child otherwise able to do so.

Table S2: Clinical definitions for suspected pneumonia, septicaemia, and meningitis

	**Age 2 to 59 months**
**Suspected pneumonia**	Suspected pneumonia is defined if there is a history of cough or difficulty breathing of less than 14 days' duration, accompanied by one or more of:<br>1. Raised respiratory rate for age[*]<br>2. Lower chest wall indrawing, nasal flaring or grunting<br>3. Oxygen saturation less than 92%<br>4. Focal chest signs (dull percussion note, coarse crackles, bronchial breathing)
**Suspected meningitis**	Suspected meningitis will be defined according to clinical judgement and is to be considered if any of the following are present:<br>1. Neck stiffness<br>2. Impaired consciousness[†]<br>3. Prostration[‡]<br>4. History of convulsion<br>5. Bulging fontanelle
**Suspected septicaemia**	Suspected septicaemia will be defined as one or more of:<br>1. Clinician diagnosis of focal sepsis (including but not limited to: septic arthritis, osteomyelitis, endocarditis, peritonitis, liver abscess, soft tissue abscess, cellulitis)<br>2. For a patient admitted, or being admitted, axillary temperature is <36°C or ≥38°C and no obvious cause of fever<br>3. For a patient admitted, or being admitted, the clinical impression is of severe malnutrition[§]

*Raised respiratory rate for age is defined as ≥ 50 breaths per minute for children at least 2 months but less than 12 months, and as ≥ 40 breaths per minute for children at least 12 months but less than 60 months.
[†]Impaired consciousness is defined as V, P, or U on the AVPU score, where A is if the patient is alert, V if responsive to verbal stimulus, P if responsive to pain stimulus, and U if unresponsive.
[‡]Prostration is defined as inability to drink or breast feed, or to remain in a seated position in a child otherwise able to do so.
[§]Severe malnutrition is defined according to the WHO definition (WHO. *Guideline: Updates on the management of severe acute malnutrition in infants and children*. Geneva: World Health Organization; 2013).

Table S3: Guideline for investigation of patients referred to clinicians and diagnosed with suspected pneumonia, septicaemia, and meningitis according to clinical definitions

a. Patients with **suspected pneumococcal disease are to have blood culture.**
b. Patients with suspected meningitis are to have lumbar puncture.
c. Patients with **suspected pneumonia are to have chest X-ray.**
d. Chest X-ray should also be considered in patients with meningitis or septicaemia if the clinician's impression is of co-existing pneumonia or if it is judged that a chest X-ray will assist in management.
e. Patients with **suspected pneumonia, septicaemia, or meningitis are to have nasopharyngeal and oropharyngeal swabs.**
f. Lung aspirate should be considered for a patient if peripheral consolidation has been demonstrated, preferably by chest X-ray.
g. Other investigations including pleural tap and joint aspirate may be considered according to the clinical indication.
h. Patients with suspected pneumococcal disease are to have,
    i. a rapid diagnostic test for malaria (January – July; only if surveillance number ends in '0').
    ii. serum collection for antibiotic activity detection if surveillance number ends in '0' or '5' and the patient is enrolled in Basse.

Table S4: Primers and probes mixtures used in the diagnostic real-time RT-PCR*

Mixture	Sequence 5'-3'	Label probe	pMol per reaction	Annealing/ elongation temp.
**EAV mix**				**60°C**
EAV-2043F	CTGTCGCTTGTGCTCAATTTAC		15	
EAV-2193R	AGCGTCCGAAGCATCTC		15	
EAV 2102P-2	TGCAGCTTATGTTCCTTGCACTGTGTTC	TXR- BHQ-2	5	
**INFAB mix**				**60°C**
INFAM-sense	AAGACCAATCCTGTCACCTCTGA		5	
INFAM-sense3	AAGACCAATCTTGTCACCTCTGA		5	
INFAM-sense4	AAGACCAATTCTGTCACCTYTGA		5	
INFAM A-sense	CAAAGCGTCTACGCTGCAGTCC		7.5	
INFAM A-sense2	TAAAGCGTCTACGCTGCAGTCC		7.5	
INFAM-probe3	TTTGTKTTCACGCTCACCGTGCC	Fam-EDQ	5	
INFB-NS779F	GTCTTAATGAAGGACATTCAAAGCC		15	
INFB-NS886R	TAAAGTTCTTCCGTGACCAGTCTA		15	
INFB 848P	GTCAAGAGCACCGATTATCACCAGAAGAG	YY-BHQ-1	5	
**RSV-A/B mix**				**55°C**
RSV A 2050F	TGAACAACCCAAAAGCATCA		15	
RSV A 2117R	CCTAGGCCAGCAGCATTG		15	
RSV A 2086P2	AATTTCCTCACTTCTCTAGTGTAGTATTGGG	FAM-BHQ-1	5	
RSV B 17 fw2	GATGGCTCTTAGCAAAGTCAAGTTGA		15	
RSV B 120 r	TGTCAATATTATCTCCTGTACTACGTTGAA		15	
RSV B PB45	TGATACATTAAATAAGGATCAGCTGCTGTCATCCA	YY-BHQ-1	5	
**H3-H1pdm09 mix**				**60°C**
H3-1541F2	CRATGTRTACAGGGATGAAGCWTTAAACA		30	
H3-1600R	TAGGATCCAATCTTTGTACCCTGACTT		15	
H3-1571P1	AGCTCAACTCCCTTGATCTGGAAYCGG	YY-BHQ-1	15	
H1-Sw-1306F	TGGACTTACAATGCCGAACT		15	
H1-Sw-1423R	CAGCCGTTTCCAATTTCCTT		15	
H1-sw-1357P2	GGACTATCACGATTCAAATGTGAAGAACT	TXR- BHQ-2	5	
**N2-N1pdm09 mix**				**60°C**
N2-278F2	GTGGCATTACAGGATTTGCAC		20	
N2-376R	CTGTCCAAGGGCAAATTGATAAC		12.5	
N2-376R1	TTGTCCAAGGGCAAATTGATAAC		12.5	
N2-327P	GACATCTGGGTGACAAGAGAACCTTAT	YY-BHQ-1	5	
N1v-1142F1	GACTGGGACTGACAATAAATTCTCAA		15	
N1v-1244R1	CAGCCCTGTTAGTTCTGGATGC		15	
N1v-1193P1	AGTGGTCAGGGTATAGCGGGAGT	FAM-BHQ-1	5	
**VIC/YAM mix**				**55°C**
INFB-HA-444F	ACCCTACARAMTTGGAACCTCAGG		18	
INFB-HA-524R	ACRGCCCAAGCCATTGTTG		12	
INFB-Yam501Pr	AAATCCGATTTTACTGGTAG	FAM-MGB-Eclipse	3	
INFB-Vic499Pr	ATCCGTTTCCATTGGTAA	ATTO532-MGB-Eclipse	2	
**PIV 1-3 mix**				**55°C**
PIV1S2	ACCTAYAAGGCAACARCATC		10	

Mixture	Sequence 5’-3’	Label probe	pMol per reaction	Annealing/ elongation temp.
PIV1As2	CTTCCTGCTGGTGTRTTAAT		10	
PIV1YY	CAAACGATGGCTGAAAAAGGGA	YY-BHQ1	5	
PIV2s	CCATTTACCTAAGTGATGGAA		10	
PIV2as2	GTRGCATAATCTTCTTTYTC		10	
PIV2TXR	AATCGCAAAACGTGTTCAGTCAC	TXR-BBQ	5	
PIV3s	CAGGRAGCATTGTGTCATCTGTC		10	
PIV3as2	TAGTGTGTAATGCAGCTYGT		10	
PIV3FAM	GTCATAACTTACTCAACAGCAAC	Fam-BHQ1	5	
**PIV 4 mix**				**55°C**
PIV4-1055F1	TAATACAATTACACTTGACTCATTAGC		10	
PIV4-1055F2	TAATACAATTACACTTGATCCGTTAGC		10	
PIV4-1156R	CCGAATCATTTCTGAKACTGTTA		10	
PIV4-1123P	GAGCAGGYGTTCGTATAGATGCAC	ATTO 465-BHQ1	5	

*Five µl RNA was added to 15 µl PCR-reagents mixture, briefly centrifuged and reverse transcription was immediately performed on heating blocs, for 15 minutes at 50°C immediately followed by 2 minutes at 95°C. After a brief centrifugation step the plates were kept cool at 4°C until they were placed in a Roche Light Cycler 480 for PCR cycling, 60 sec 95°C, 50 cycles of 10 sec 95°C followed by 30 sec at 40°C.

Table S5. Virus strains used as positive control in the real-time RT-PCR

Strain	RT-PCR mix
Equine arteritis virus culture 27-03-2006	EAV mix
Influenza virus A/Netherlands/1250/2016 H1N1pdm09 P3 MDCK 30/6-4/7/16	INFAB mix, H3-H1pdm09 mix, N2-N1pdm09 mix
Influenza virus A/Netherlands/379/2016 H3N2 P3 MDCK 16/6-23/6/16	INFAB mix, H3-H1pdm09 mix, N2-N1pdm09 mix
Influenza virus B/Netherlands/1515/2016 Victoria-lineage P2 MDCK 15/3-17/3/16	INFAB mix, VIC/YAM mix
Influenza virus B/Netherlands/365/2016 Yamagata-lineage P3 MDCK 22/8-24/8/16	INFAB mix, VIC/YAM mix
RSV-A 17 P4 HEP2	RSV-A/B mix
RSV-B 25 P4 HEP2	RSV-A/B mix
PIV1 2836 Strain C35 ATCC VR-94	PIV 1-3 mix
PIV2 4412 Strain ‘Greer’ ATCC VR-92	PIV 1-3 mix
PIV3 2838 Strain C243 ATCC VR-93	PIV 1-3 mix
PIV4 2839 type 4A strain M-25 ATCC VR-279	PIV 4 mix

Sequencing full length influenza virus hemagglutinin and partial RSV G- and F-protein genes

Influenza viruses

We extracted RNA from clinical specimens as described for the diagnostic RT-PCRs in the main manuscript.

Influenza virus type A cDNA was synthesized from extracted RNA using the following assay setup:

**Component**	**µl**
H_2O	1.875
Superscript III RT Buffer 5x (Thermo Fisher)	5
dNTPs 10 mM each	2.5
DTT 0.1 M	2.5
cDNA primer (Table S6) 20 pmol/µl	2
RNasin RNase Inhibitor 40 U/µl (Promega)	0.625
Superscript III Reverse transcriptase 200 U/µl (Thermo Fisher)	0.5
Specimen RNA	10
Total volume	25

60 minutes 42°C, 5 minutes 95°C, cool down and store at 4°C until amplification.

Subsequently, amplification was performed for HA1 and HA2 separately using the following assay setup:

**Component**	**µl A(H1N1)pdm09**	**µl A(H3N2)**
H_2O	7.37	19.75
Kapa Long Range buffer 5x	5	10
MgCl2 25 mM	1.75	3.5
dNTPs 10 mM each	0.75	1.5
Forward primer (Table S6) 5 pmol/µl	2.5	5
Reverse primer (Table S6) 5 pmol/µl	2.5	5
Kapa Long Range DNA Polymerase 5 U/µl	0.13	0.25
cDNA	5	5
Total volume	25	50

2 minutes 94°C, 40 cycles of 20 seconds 94°C, 15 seconds 55°C and 2 minutes 68°C, 2 minutes 72°C, cool down and store at 4°C until sequencing.

Influenza virus type B cDNA was synthesized from extracted RNA using the following assay setup:

**Component**	**µl**
RNA	23
cDNA primer (Table S7) 20 pmol/µl	1
dNTPs 10 mM each	2
RNasin RNase Inhibitor 40 U/µl (Promega)	1
**5 min at 65°C, cool on ice**	
Superscript III RT Buffer 5x (ThermoFisher)	8
DTT 0.1 M	2
RNasin RNase Inhibitor 40 U/µl (Promega)	1
H_2O	1
Superscript III Reverse transcriptase 200 U/µl (ThermoFisher)	1
Total volume	40

60 minutes 50°C, 5 minutes 95°C cool down and store at 4°C until amplification.

Subsequently, amplification was performed for HA1 and HA2 separately using the following assay setup:

**Component**	**µl**
H_2O	31.5
QIAGEN 10x HotStarTaq plus PCR buffer	5
MgCl2 25 mM	2
dNTPs 10 mM each	1
Forward primer (Table S7) 5 pmol/µl	4
Reverse primer (Table S7) 5 pmol/µl	4
QIAGEN HotStarTaq plus 5 U/µl	0.5
cDNA	2
Total volume	50

10 minutes 95°C; 35 cycles of 60 seconds 95°C, 60 seconds 45°C and 90 seconds 72°C; 10 minutes 72°C, cool down and store at 4°C until sequencing

Prior to sequencing, the amplificate was cleaned using ExoSAP-IT reagent (Thermo Fisher). Four µl ExoSAP-IT reagent was mixed with 10 µl PCR product and incubated 15 minutes 37°C, 15 minutes 80°C, cooled down and stored at 4°C until further processing.

For sequencing, cleaned PCR product was mixed at 10 ng/100 nucleotides with 20 pmol (4 µl) of the forward or reverse sequencing primer (Tables S6 and S7) and filled up to 20 µl with H_2O.

The sealed microtiter plate was submitted to the sequence service provider BaseClear for Sanger sequencing using the 96-WELL PREMIX SEQUENCING SERVICE (http://www.baseclear.com/genomics/sanger-sequencing/96-well-plates). BaseClear returns the raw sequencing trace files for further processing. The trace files were analysed and assembled in one consensus sequence per specimen in BioNumerics version 7.6.2.

Table S6. Primers for sequencing the full length hemagglutinin gene of type A influenza viruses

Name	Primer 5'- 3'	Used for
**Generic type A**		
Uni12	AGCRAAAAGCAGG	cDNA
**A(H1N1)pdm09**		
Amplification		
HAFM	CGAGGAGCAAAAGCAGGGG	HA1
H1v 1412R	TTTAGCTGGCTTCTTACCT	HA1
H1v 679F	TTCAAGCCGGAAATAGCAATAAG	HA2
HARUc	ATATCGTCTCGTATTAGTAGAAACAAGGGTGTTTT	HA2
Sequencing		
HAFM	CGAGGAGCAAAAGCAGGGG	HA1
H1-Mex 416F	CATGGCCCAATCATGACTC	HA1
H1v 785R2	GTTGCTTCGAATGTTATTTTG	HA1
H1-Mex 997R	CGGGATATTCCTCAATCCTGT	HA1
H1-Mex 1301F	ACATTTGGACTTACAATGCCGAA	HA2
H1-Mex 679F	TTCAAGCCGGAAATAGCAATAAG	HA2
H1 Mex 1609R	GTACCAATGAACTGGCGAC	HA2
HARUc	ATATCGTCTCGTATTAGTAGAAACAAGGGTGTTTT	HA2
**A(H3N2)**		
Amplification		
HAFM	CGAGGAGCAAAAGCAGGGG	HA1
H3-1053R	TTGCGCCAAATATGCCTCTA	HA1
H3-498-11F	GTGACTATGCCRAACAATGAAC	HA2
HAR K	GTATTAGTAGAAACAAGGGTGTTTT	HA2
Sequencing		
HAFM	CGAGGAGCAAAAGCAGGGG	HA1
H3-498-11 Fw	GTGACTATGCCRAACAATGAAC	HA1
H3-600R	GGTAGAKACTGTGATTCTTC	HA1
H3-1053R	TTGCGCCAAATATGCCTCTA	HA1
H3-498-11 Fw	GTGACTATGCCRAACAATGAAC	HA2
H3-960F	AAACAGGATCACATACGGG	HA2
H3-1020F	GGCGCAATAGCKGGTTTCATAG	HA2
H3-1053R	TTGCGCCAAATATGCCTCTA	HA2
HAR K	GTATTAGTAGAAACAAGGGTGTTTT	HA2

Table S7. Primers for sequencing the full length hemagglutinin gene of type B influenza viruses

Name	Primer 5'- 3'	Used for
cDNA synthesis		
BHA1-new	AAGCAGAGCATCTTCTCAAAACTGA	cDNA HA
Amplification		
infB Ha fwd1	GCAGAGCATTTTCTAATATCCACAA	HA1
infB Ha rev3	GCTTTTGTTTAATCCACCCGTATTT	HA1
infB Ha fwd4	GCCTTTAATTGGAGAAGCAGATTG	HA2
infB Ha rev6	TTTTCAATAACGTTTCTTTGTAATG	HA2
Sequencing		
infB Ha fwd1	GCAGAGCATTTTCTAATATCCACAA	HA1
infB Ha rev1	TTTTTGTTCTGTCGTGCATTATAG	HA1
infB Ha fwd2	TTCAATACTCCATGAAGTCAGACC	HA1
infB Ha rev2	GGCTTTGAGTCCCCATAGAG	HA1
infB Ha fwd3	CATTTGTACAGAAGGAGAAGACCA	HA1
infB Ha rev3	GCTTTTGTTTAATCCACCGTATTT	HA1
infB Ha fwd4	GCCTTTAATTGGAGAAGCAGATTG	HA2
infB Ha rev4	CATGGCACCGCTTAGTCTT	HA2
infB Ha fwd5	CAAGAGGCCATAAACAAGATAACA	HA2
infB Ha rev5	TGAATCAAAGGTGGGGAGAG	HA2
infB Ha fwd6	CTTGAAAGAAAGCTGAAGAAAATG	HA2
infB Ha rev6	GCCTTTAATTGGAGAAGCAGATTG	HA2

Respiratory syncytial virus

RSVA/B G-protein gene sequencing

cDNA synthesis and first amplification from extracted RNA was performed using the following one-step assay setup:

Component	µl
H_2O	28.5
QIAGEN OneStep RT-PCR Buffer 5x	10
QIAGEN OneStep RT-PCR Kit enzyme mix	2
dNTPs 10 mM each	2
cDNA and forward primer 20 pmol/µl (Table S8)	1
Reverse primer 20 pmol/µl (Table S8)	1
RNase OUT RNase Inhibitor 40 U/µl (ThermoFisher)	0.5
Specimen RNA	5
Total volume	50

cDNA step: 30 minutes 50°C,15 minutes 95°C.
PCR step: 40 cycles of 30 seconds 94°C, 30 seconds 54°C and 60 seconds 72°C, 10 minutes 72°C.

Nested amplification of first amplification product was performed using the following assay setup:

**Component**	**µl**
H_2O	21
QIAGEN HotStarTaq Master Mix	25
Forward primer 20 pmol/µl (Table S8)	1
Reverse primer 20 pmol/µl (Table S8)	1
First amplification product	2
Total volume	50

15 minutes 95°C, 30 cycles of 45 seconds 94°C, 45 seconds 54°C and 60 seconds 72°C, 10 minutes 72°C.

Prior to sequencing, the amplificate was cleaned using ExoSAP-IT reagent (ThermoFisher). Four µl ExoSAP-IT reagent was mixed with 10 µl PCR product and incubated 15 minutes 37°C, 15 minutes 80°C, cooled down and stored at 4°C until further processing.

For sequencing, cleaned PCR product was mixed at 10 ng/100 nucleotides with 20 pmol (4 µl) of the forward or reverse sequencing primer (Table S8) and filled up to 20 µl with H_2O. The sealed microtiter plate was submitted to BaseClear for Sanger sequencing using the 96-WELL PREMIX SEQUENCING SERVICE (http://www.baseclear.com/genomics/sanger-sequencing/96-well-plates). BaseClear returns the raw sequencing trace files for further processing. The trace files were analysed and assembled in one consensus sequence per specimen in BioNumerics version 7.6.2.

Table S8. Primers for sequencing the partial G-protein genes of RSV*

**Name**	**Primer 5'- 3'**	**Used for**
**One-step cDNA synthesis and first round amplification**		
AG20F	GGGGCAAATGCAAMCATGTCC	cDNA and PCR
F164R1	GTTATGACACTGGTATACCAACC	PCR
F164R2	GTTATAACACTAGTATACCAACC	PCR
**Nested amplification and sequencing**		
BG10F	GCAATGATAATCTCAACYTC	Nested PCR and sequencing A & B
F1R1	CAACTCCATTGTTATTTGCC	Nested PCR and sequencing A
F1R2	CAACKCCATGGTTATTTGCC	Nested PCR and sequencing B
G523F	ATATGCAGCAACAATCCAAC	Sequencing A
G523R	GTTGGATTGTTGCTGCATAT	Sequencing A
G533F	TGTAGTATATGTGGCAACAA	Sequencing B
G533R	TTGTTGCCACATATACTACA	Sequencing B

* Adapted from: Agoti CN, Mwihuri AG, Sande CJ, Onyango CO, Medley GF, Cane PA, Nokes DJ. Genetic relatedness of infecting and reinfecting respiratory syncytial virus strains identified in a birth cohort from rural Kenya. J Infect Dis. 2012 Nov 15;206(10):1532-41. The partial sequence ranges from halfway the transmembrane region amino acid position 54 through the whole external part of the G protein up to the stop codon. A number of sequences are extended due to the loss of one or more stop codons. These are indicated in the phylogenetic trees with the number of nucleotides in the extension.

RSV A/B F-protein gene sequencing

cDNA synthesis was performed using the following assay setup:

**cDNA Primer mix**	
**Component**	**µl**
H_2O	1
Random hexamers (ThermoFisher) 50 ng/µl	1
dNTPs 10 mM each	1
Specimen RNA	10
Total volume	13

5 minutes 65°C, cool down for 2 minutes, spin briefly and add 7 µl of the following enzyme mix:

**Enzyme mix**	
**Component**	**µl**
Superscript III RT Buffer 5x (ThermoFisher)	4
DTT 0.1 M	1
RNasin RNase Inhibitor 40 U/µl	1
Superscript III Reverse transcriptase 200 U/µl (ThermoFisher)	1
Total volume	7

Mix briefly and incubate for 5 minutes at 25°C followed by 60 minutes 50°C.

First amplification from cDNA for RSV-A F was performed using the following assay setup:

**Component**	**µl**
H_2O	14.75
Kapa Long Range buffer 5x	10
MgCl2 25 mM	3.5
dNTPs 10 mM each	1.5
Forward primer (Table S9) 5 pmol/µl RSVA-5648-S	5
Reverse primer (Table S9) 5 pmol/µl RSVA-7418-AN	5
Kapa Long Range DNA Polymerase 5U/µl	0.25
cDNA	10
Total volume	50

120 seconds 94°C; 35 cycles of 30 seconds 94°C, 30 seconds 52°C and 120 seconds 68°C ;10 minutes 68°C.

Nested amplification of first amplification product for RSV-A F was performed using the following assay setup:

**Component**	**µl**
H_2O	6
QIAGEN HotStarTaq Master Mix	12.5
Forward primer (Table S9) 5 pmol/µl RSVA-5648-S or RSVA-6366-F	2.5
Reverse primer (Table S9) 5 pmol/µl RSVA-6611-AN or RSVAB-6761-R	2.5
First amplification product	1.5
Total volume	25

15 minutes 95°C, 30 cycles of 30 seconds 94°C, 30 seconds 52°C and 30 seconds 72°C, 10 minutes 72°C.

First amplification from cDNA for RSV-B F was performed using the following assay setup:

**Component**	**µl**
H_2O	14.75
Kapa Long Range buffer 5x	10
MgCl2 25 mM	3.5
dNTPs 10 mM each	1.5
Forward primer (Table S9) 5 pmol/µl RSVB-5502-S	5
Reverse primer (Table S9) 5 pmol/µl RSVB-7402-AN	5
Kapa Long Range DNA Polymerase 5 U/µl	0.25
cDNA	10
Total volume	50

120 seconds 94°C; 35 cycles of 30 seconds 94°C, 30 seconds 52°C and 120 seconds 68°C ;10 minutes 68°C.

Nested amplification of first amplification product for RSV-B F was performed using the following assay setup:

**Component**	**µl**
H_2O	6
QIAGEN HotStarTaq Master Mix	12.5
Forward primer (Table S9) 5 pmol/µl RSVB-5502-S or RSVB-5828-R	2.5
Reverse primer (Table S9) 5 pmol/µl RSVB-6285-R or RSV AB-6761-R	2.5
First amplification product	1.5
Total volume	25

15 minutes 95°C, 30 cycles of 30 seconds 94°C, 30 seconds 52°C and 30 seconds 72°C, 10 minutes 72°C.

Prior to sequencing, the amplificate was cleaned using ExoSAP-IT reagent (ThermoFisher). Four µl ExoSAP-IT reagent was mixed with 10 µl PCR product and incubated 15 minutes 37°C, 15 minutes 80°C, cooled down and stored at 4°C until further processing.

For sequencing, cleaned PCR product was mixed at 10 ng/100 nucleotides with 20 pmol (4 µl) of the forward or reverse sequencing primer (Table S9) and filled up to 20 µl with H_2O. The sealed microtiter plate was submitted to BaseClear for Sanger sequencing using the 96-WELL PREMIX SEQUENCING SERVICE (http://www.baseclear.com/genomics/sanger-

sequencing/96-well-plates). BaseClear company returns the raw sequencing trace files for further processing. The trace files were analysed and assembled in one consensus sequence per specimen in BioNumerics version 7.6.2.

Table S9. Primers for sequencing the partial F-protein genes of RSV

Name	Primer 5'- 3'	Used for
**First round amplification**		
RSVA-5648-S	GGGGCAAATAACAATGGAGTT*	PCR
RSVA-7418-AN	CATTGTAAGAACATGATTAGGTGCT*	PCR
RSVB-5502-S	CGAAAACACACCCAACTCCACAC*	PCR
RSVB-7402-AN	GTGGTTTTTTGTCTATTTGCTG*	PCR
**Nested amplification and sequencing**		
RSVA-5648-S	GGGGCAAATAACAATGGAGTT*	Nested PCR and sequencing
RSVA-6611-AN	GTGTAGTTTCCAACAAGGAG*	Nested PCR and sequencing
RSVA-6366-F	AATTTAGTGTYAATGCAGGTGTAACTAC	Nested PCR and sequencing
RSVAB-6761-R	TGGTAATGTYAAACTGTTCATTGTGTC	Nested PCR and sequencing
RSVA-5942-F	AGTTGCTCATGCAAAGCACAC	Sequencing
RSVA-6595-R	GTGTGTAGTTTCCAACAAGG	Sequencing
RSVB-5502-S	CGAAAACACACCCAACTCCACAC*	Nested PCR and sequencing
RSVB-6285-R	AGATCTAACACTTTGCTGGTTAA	Nested PCR and sequencing
RSVAB-6761-R	TGGTAATGTYAAACTGTTCATTGTGTC	Nested PCR and sequencing
RSVB-6555-R	CTTTGTTGCCTTACTATCTGAACA	Sequencing

* Adapted from: Xia Q, Zhou L, Peng C, Hao R, Ni K, Zang N, Ren L, Deng Y, Xie X, He L, Tian D, Wang L, Huang A, Zhao Y, Zhao X, Fu Z, Tu W, Liu E. Detection of respiratory syncytial virus fusion protein variants between 2009 and 2012 in China. Arch Virol. 2014 May;159(5):1089-98.

Table S10. Clinical characteristics of a representative sample, and all children, aged 2-23 months with acute lower respiratory infection from February to December 2015 in the BHDSS, rural Gambia

Characteristic	Randomly sampled group (n=519)	Group not sampled (n=1866)
Mean age in days (SD)	10.8 (6.2)	10.8 (6.2)
Female	236 (45.5%)	813 (43.6%)
Mean respiratory rate (SD)	55 (10)	56 (11)
Lower chest wall indrawing	264 (50.9%)	960 (51.5%)
Wheeze on auscultation	150 (28.9%)	520 (27.9%)
O_2 saturation <93%	45 (8.7%)	134 (7.2%)
Radiological pneumonia	52 (10.0%)	201 (10.8%)
Bacteremia	8 (1.5%)	39 (2.1%)
Hospitalised	291 (56.1%)	1057 (56.7%)
Death	3 (0.6%)	24 (1.3%)

Table S11: Monthly numbers of: ALRI cases, cases sampled for viral testing and viral-associated ALRI, from February to December 2015 among children aged 2-23 months in the BHDSS, rural Gambia

	Number sampled / Number of ALRI cases										
	February (49/192)	March (42/290)	April (48/278)	May (48/199)	June (42/217)	July (55/186)	August (52/305)	September (48/287)	October (41/203)	November (52/142)	December (42/86)
	Number of observed viral-associated ALRI cases										
Any virus	33	31	34	27	26	29	42	40	29	7	5
RSV-A	31	30	29	20	13	26	39	32	13	4	1
RSV-B	0	0	1	1	1	1	1	0	2	0	0
Inf-A	0	1	1	2	0	0	0	4	10	1	0
Inf-B	0	0	0	2	2	1	1	9	4	1	0
PIV1	0	0	0	0	0	0	0	0	1	0	0
PIV3	2	0	4	3	5	0	0	1	0	0	1
PIV4	1	0	0	0	5	1	2	1	1	2	3

Table S12: Monthly numbers of: ALRI cases, cases sampled for viral testing and viral-associated ALRI, from February to December 2015 among children aged 2-11 months in the BHDSS, rural Gambia

	Number sampled / Number of ALRI cases										
	February (30/116)	March (32/189)	April (33/203)	May (24/124)	June (29/126)	July (34/117)	August (29/182)	September (29/157)	October 19/108)	November (34/76)	December (28/48)
	Number of observed viral-associated ALRI cases										
Any virus	25	23	24	15	18	17	26	24	15	5	4
RSV-A	25	23	22	11	10	15	25	20	9	4	1
RSV-B	0	0	0	1	0	1	0	0	1	0	0
Inf-A	0	0	1	0	0	0	0	3	5	0	0
Inf-B	0	0	0	1	1	0	0	4	0	0	0
PIV1	0	0	0	0	0	0	0	0	1	0	0
PIV3	0	0	2	2	4	0	0	1	0	0	1
PIV4	1	0	0	0	3	1	1	0	0	2	2

Table S13: Monthly numbers of: ALRI cases, cases sampled for viral testing and viral-associated ALRI, from February to December 2015 among children aged 12-23 months in the BHDSS, rural Gambia

	Number sampled / Number of ALRI cases										
	February (19/76)	March (10/101)	April (15/75)	May (24/75)	June (13/91)	July (21/69)	August (23/123)	September (19/130)	October (22/95)	November (18/66)	December (14/38)
	Number of observed viral-associated ALRI cases										
Any virus	8	8	10	12	8	12	16	16	14	2	1
RSV-A	6	7	7	9	3	11	14	12	4	0	0
RSV-B	0	0	1	0	1	0	1	0	1	0	0
Inf-A	0	1	0	2	0	0	0	1	5	1	0
Inf-B	0	0	0	1	1	1	1	5	4	1	0
PIV1	0	0	0	0	0	0	0	0	0	0	0
PIV3	2	0	2	1	1	0	0	0	0	0	0
PIV4	0	0	0	0	2	0	1	1	1	0	1

Simulation of the expected number of cases in the population taking into account the monthly sampling scheme

An example of one simulation (PIV1, 2-11 mo age group; Table S12) is given in the R code below.

nsamp is the number of times (1,000,000 in the study) that the sampling exercise is run in order to generate the estimated number of test-positives in the untested cohort with ALRI.

p represents the number of observed cases per month divided by the number of patients randomly selected for viral testing per month.

n represents the number of randomly selected patients per month.

N represents the total number of patients with ALRI per month.

```
simulate_sampling=function(nsamp){
set.seed(227083);
M=11; # Number of months
p=cbind(0/30,0/32,0/33,0/24,0/29,0/34,0/29,0/29,1/19,0/34,0/28)
n=cbind(30,32,33,24,29,34,29,29,19,34,28)
N=cbind(116,189,203,124,126,117,182,157,108,76,48)

# Simulation
s=array(0, c(M,nsamp)) # array of months * 1 col for each simualtion
pp=array(0,nsamp)# These will hold the simulated probs
for (i in 1 : M){
   pp = rbinom(nsamp,n[i],p[i])/n[i];
   for (j in 1 : nsamp){
      s[i,j]=rbinom(1,N[i],pp[j])
   }
}
t=colSums(s);
lower=quantile(t,0.025);
upper=quantile(t,0.975);

# Normal approximation
pave = mean(p);
pvar = (1/(M^2))*sum(p*(1-p)/n);
Nlower95ci = sum(N)*(pave-1.96*sqrt(pvar));
Nupper95ci = sum(N)*(pave+1.96*sqrt(pvar));
NexpN = pave*sum(N);

# Lower and upper 95 CI and the expected overall count, for simulation and normal approx
list(lower95CI = lower,
upper95CI = upper,
expN = mean(t),
Nlower95ci=Nlower95ci,
Nupper95ci=Nupper95ci,
NexpN=NexpN)
}
```

Phylogenetic analysis

Phylogenetic trees annotated with amino acid substitutions have been inferred from the generated influenza virus hemagglutinin and RSV G-protein gene sequences using the protein coding parts, devoid of signal peptide and stop codon. First the sequences were aligned using BioEdit software version 7.2.5 [1] with ClustalW algorithm [2] and manually refined taking into account the right reading frame for translation and subsequently trimmed to remove signal peptide and stop codon. The annotated phylogenetic analysis was executed by making use of the treesub package (https://github.com/tamuri/treesub) which combines Maximum Likelihood methodology of RAxML 8.2.10 [3], and the annotated tree inference and branch length estimation of the baseml function in PAML 4.9 [4]. At first a maximum-likelihood analysis was performed by fitting to the GTRGAMMA model. Subsequently bootstrapping was done using the autoMRE (majority-rule extended) function of RAxML which performs bootstraps until convergence [5]. The optimal tree, as indicated by RAxML, was provided of the respective amino acid substitutions by baseml as called by treesub. FigTree 1.4.3 [6] was used to visualise the trees, pre-prepare for publication and export in pdf format. Adobe Illustrator CC 2017 software was used to add the bootstrap values taking them from the bootstrapped tree visualised in FigTree. For influenza virus the Gambian influenza viruses are set in the context of recent vaccine strains and viruses from around the world and specifically from the African continent and from the Netherlands from the same sampling period representing recent genetic groups circulating. For RSV the Gambian RSV were set in the context of viruses from around the world and specifically from the Netherlands from the same sampling period representing the recent diversity of genetic groups circulating. Sequences covering the stretch of the RSV G-protein gene used in our study were not available for viruses from the African continent sampled in the same period as the Gambian RSV when sequences for our analysis were downloaded from GenBank in January 2018. Nevertheless, we used shorter sequences of Kenyan RSV available in January 2018 covering most of the ectodomain coding part of the G-protein gene sampled in the same time period as our Gambian RSV. We conducted a preliminary phylogenetic analysis (Neighbor-Joining with Jukes-Cantor model, pairwise deletion of missing sequence parts and 1000 bootstraps in MEGA version 7) to see whether Gambian and Kenyan RSV from 2015 segregate similarly with RSV from other continents (Figure S1). Kenyan sequences for this analysis were retrieved from Genbank PopSet 1153279097 for RSV-A[7] and 1071930237 for RSV-B[8]. This preliminary analysis used the same set of other Kenyan RSV sequences from before 2014 and other continent RSV with more complete G-protein sequences that we used for our full analysis. Accession numbers of all sequences downloaded from GenBank and GISAID databases and used in the phylogenetic analysis are listed in Table S14. Sequences of partial G protein genes of RSV and full length hemagglutinin gene segment of influenza viruses from patients in The Gambia generated in this study and sequences of partial G protein gene of RSV from patients in the Netherlands not previously reported have the following accession numbers: for RSV G protein genes from Gambian patients: GenBank MG971446-MG971464; for RSV G protein genes from Dutch patients: Genbank MG971421-MG971445; for influenza viruses from The Gambia: GISAID EPI1180580- EPI1180595. The names of viruses from The Gambia have been color coded in the phylogenetic trees in Figures S2-S5 to indicate the month of sampling in 2015. Amino acid substitutions relative to the oldest vaccine virus included for influenza viruses and oldest strain included for RSV are indicated on or below branches after which the allocated viruses have the specified amino acid substitution in common. The amino acid numbering follows that of the consensus sequence after alignment of the included sequences taking into account the start of the coding sequence for partial sequences and all insertions/deletions and extensions due to loss of stop codons.

Potential N-glycosylation sites on the HA protein of influenza virus and the G protein of RSV and potential O-glycosylation sites on the G protein of RSV were predicted using the NetNGlyc server version 1.0 (http://www.cbs.dtu.dk/services/NetNGlyc/) and the NetOGlyc server version 4.0 (http://www.cbs.dtu.dk/services/NetOGlyc/) respectively. Predicted sites are superimposed on the phylogenetic tree: -N or +N indicate the loss or gain of an N-glycosylation site respectively; -O or +O indicate the loss or gain of an O-glycosylation site respectively. Insertions are indicated with ▼, deletions with ▲. The percentage of replicate trees in which the associated taxa clustered together in the bootstrap test is shown on or below the branches for values ≥ 70%.

1. Hall, T.A. BioEdit: a user-friendly biological sequence alignment editor and analysis program for Windows 95/98/NT. Nucl. Acids. Symp. Ser. 1999;41:95-98.
2. Thompson JD, Higgins DG, Gibson TJ. CLUSTAL W: improving the sensitivity of progressive multiple sequence alignment through sequence weighting, position-specific gap penalties and weight matrix choice. Nucleic Acids Res. 1994 Nov 11;22(22):4673-80.
3. Stamatakis A. RAxML version 8: a tool for phylogenetic analysis and post-analysis of large phylogenies. Bioinformatics. 2014 May 1;30(9):1312-3.
4. Yang Z. PAML 4: phylogenetic analysis by maximum likelihood. Mol Biol Evol. 2007 Aug;24(8):1586-91.
5. Pattengale ND, Alipour M, Bininda-Emonds OR, Moret BM, Stamatakis A. How many bootstrap replicates are necessary? J Comput Biol. 2010 Mar;17(3):337-54.
6. Rambaut A. FigTree tree figure drawing tool version 1.4.3. 2006-2016, Institute of Evolutionary Biology, University of Edinburgh. http://tree.bio.ed.ac.uk/
7. Otieno JR, Kamau EM, Agoti CN, Lewa C, Otieno G, Bett A, Ngama M, Cane PA, Nokes DJ. Spread and Evolution of Respiratory Syncytial Virus A Genotype ON1, Coastal Kenya, 2010-2015. Emerg Infect Dis. 2017 Feb;23(2):264-271.
8. Kamau,E.M., Agoti,C.N., Lewa,C.S., Cane,P.A., Bett,A., Medley,G.F. and Nokes,J.D. Local and global long-term trends in evolution of attachment (G) gene of RSV genotype BA. Unpublished; Direct Submission GenBank. Released 5 October 2016.

Table S14. Accesssion numbers for RSV G protein gene sequences retrieved from GenBank and for influenza virus hemagglutinin gene sequences from GISAID (downloaded January 2018). All submitters are highly acknowledged for sharing of sequences.

RSV-A	RSV-B	A(H1N1)pdm09	A(H3N2)	B/Yamagata
AY911262.1	KU316116.1	EPI239901	EPI359498	EPI159978
KM042383.1	KU950534.1	EPI849370	EPI407123	EPI328916
KY982516.1	KU950565.1	EPI278560	EPI696955	EPI418115
KJ672469.1	KU950672.1	EPI678532	EPI614406	EPI539767
KU950673.1	KU950689.1	EPI624673	EPI824058	EPI533306
KX655640.1	KX655654.1	EPI539472	EPI773595	EPI590295
KX765886.1	KY249658.1	EPI574439	EPI781616	EPI590296
KY654514.1	KU950477.1	EPI829401	EPI539794	EPI590305
KU839630.1	KX765927.1	EPI319473	EPI466762	EPI590307
KY654518.1	KJ627262.1	EPI770123	EPI541428	EPI590311
KX765944.1	KY249671.1	EPI279891	EPI466774	EPI590313
KX765941.1	KX655674.1	EPI279893	EPI630731	EPI590317
KX765970.1	KY249663.1	EPI536263	EPI426061	EPI590319
KU950523.1	KU950461.1	EPI697729	EPI352771	EPI844477
KY654517.1	KX655642.1	EPI286992	EPI392272	EPI540653
KX655626.1	KX765952.1	EPI379969	EPI590349	EPI539438

RSV-A	RSV-B	A(H1N1)pdm09	A(H3N2)	B/Yamagata
KJ627264.1	KF826858.1	EPI390483	EPI590370	EPI541113
KX765955.1	KP317928.1	EPI331210	EPI590373	EPI377694
KX765911.1	KU950588.1	EPI731589	EPI873251	EPI467167
KX655677.1	KX765968.1	EPI590367	EPI878333	EPI319558
KX765960.1	JX576729.1	EPI590355	EPI878325	EPI504820
KJ627374.1	KF530266.1	EPI731625	EPI878329	EPI737991
KU950546.1	KF826860.1	EPI731633	EPI885210	EPI738007
KJ939936.1	KJ939932.1	EPI590361	EPI590364	EPI744889
KX655627.1	KX765905.1	EPI624730	EPI731539	EPI708747
KX655689.1	KY249667.1	EPI825154	EPI829339	EPI708755
KJ627296.1	KX655653.1	EPI334730	EPI466802	EPI737662
KJ939962.1	KU950467.1	EPI379577	EPI630809	EPI737678
KF826856.1	KX765959.1	EPI346697	EPI829365	EPI630534
KX510211.1	KY249660.1	EPI316435	EPI630823	EPI744905
KP317953.1	KX765906.1	EPI733766	EPI552698	EPI693058
KX510151.1	KY249683.1	EPI756377	EPI729946	EPI693025
KF826840.1	KF826857.1	PI1020050	EPI747069	EPI574770
KP317924.1	KF826839.1	EPI630606	EPI746007	EPI717291
KP317950.1	KF826844.1	EPI706886	EPI746011	EPI710646
KJ627339.1	KX655685.1	EPI748870	EPI746017	EPI696829
MG642081.1	KY249661.1	EPI748876	EPI746021	EPI730810
KU316138.1	KX765923.1	EPI733203	EPI730050	EPI649359
KP258725.1	KU950489.1	EPI742364	EPI730082	EPI678873
KU316145.1	KP317945.1	EPI706391	EPI630692	EPI693299
KF973333.1	KP317946.1	EPI630616	EPI730927	EPI675648
KF826836.1	KP317932.1	EPI630622	EPI624553	EPI693222
KP317956.1	MG431252.1	EPI748890	EPI704211	EPI737564
KJ643504.1	JX576760.1	EPI756426	EPI778036	EPI721111
KU316168.1	KP258742.1	EPI756434	EPI710225	EPI731456
KU316169.1	KF826853.2	EPI733780	EPI688645	EPI753859
	KP317923.1	EPI718183	EPI688715	EPI753881
	KP317939.1	EPI691342	EPI688878	
	KP258720.1	EPI729903	EPI688870	
	KU316114.1	EPI649341	EPI729904	
	KU316108.1	EPI678954	EPI672371	
	MG642057.1	EPI684386	EPI769499	
	KU316144.1	EPI684394	EPI693663	
		EPI630676	EPI710313	
		EPI630684	EPI746402	
		EPI732982	EPI684299	
		EPI716187	EPI710409	
		EPI756119	EPI769549	
		EPI756127	EPI752115	
		EPI733884	EPI672327	

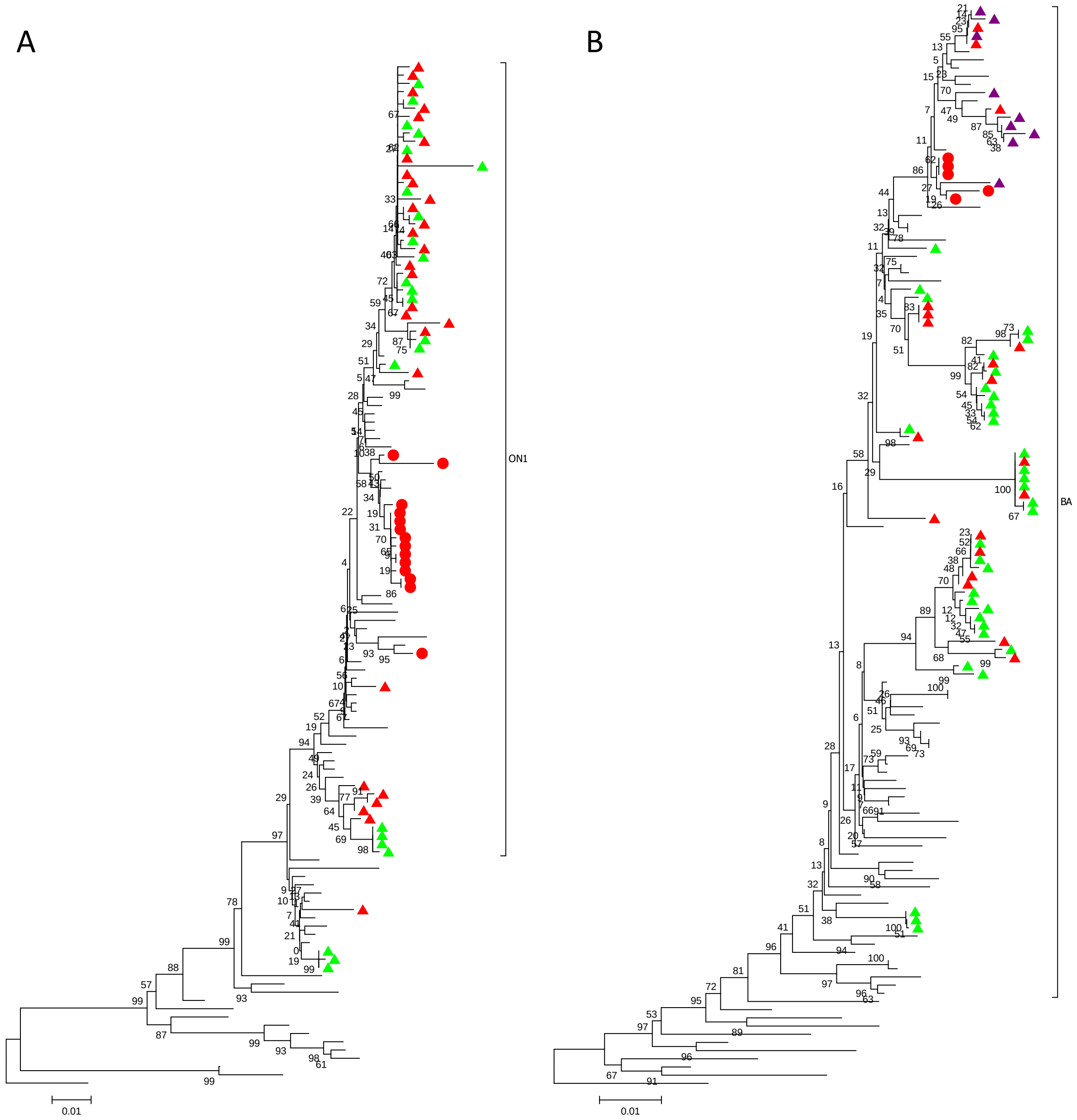

Figure S1. Preliminary phylogenetic analysis of G-protein coding sequence to show possible correlation between Gambian and Kenyan RSV collected in 2015.

A) RSV-A. Coding sequences 120 nucleotides shorter for 2014 and 2015 Kenyan RSV-A compared to RSV-A G-protein gene sequences with complete ectodomain from The Gambia 2015, Kenya before 2014 and other countries on other continents. B) RSV-B. Coding sequences 45-105 nucleotides shorter for 2014, 2015 and 2016 Kenyan RSV-B compared to RSV-B G-protein gene sequences with complete ectodomain from The Gambia 2015, Kenya before 2014 and other countries on other continents. Green = 2014; Red = 2015; Purple = 2016. Circle = Gambian RSV; Triangle = Kenyan RSV; No Label = RSV from Kenya before 2014 or from other countries on other continents of which the placement relative to the Gambian 2015 RSV is shown in Figure 2 (RSV-A) and Figure S2 (RSV-B). Bootstrap values are shown on the branches.

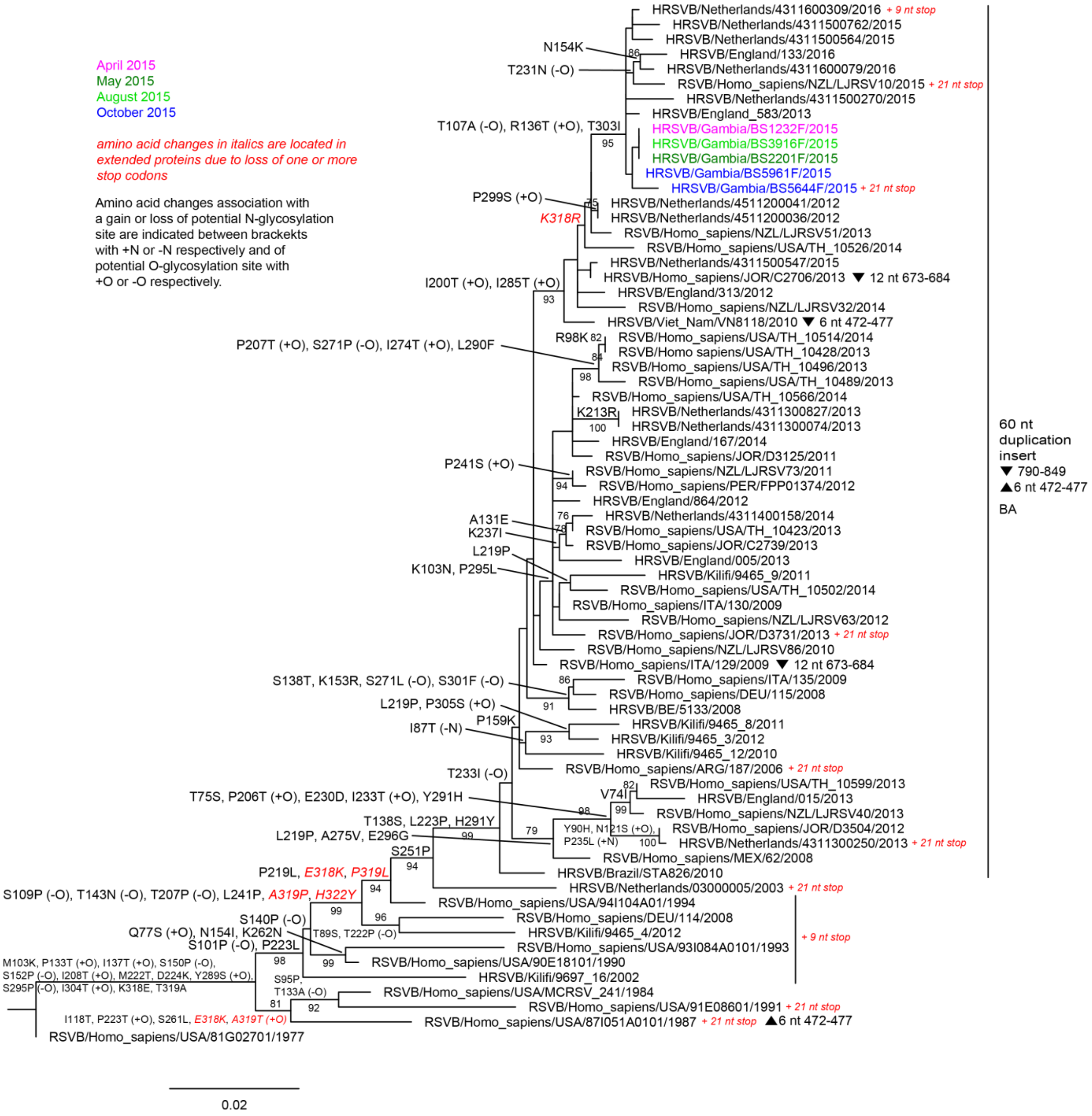

Note: country codes in the virus names: ARG=Argentina; BE=Belgium; DEU=Germany; ITA=Italy; JOR=Jordan; Kilifi=Kenya; MEX=Mexico; NZL=New Zealand; PER=Peru; USA=United States of America

Figure S2. Phylogenetic tree for RSV-B, based on partial sequences of the G-protein gene. Protein sequence ranges from amino acid position 54 located in the transmembrane part up to the stop codon.

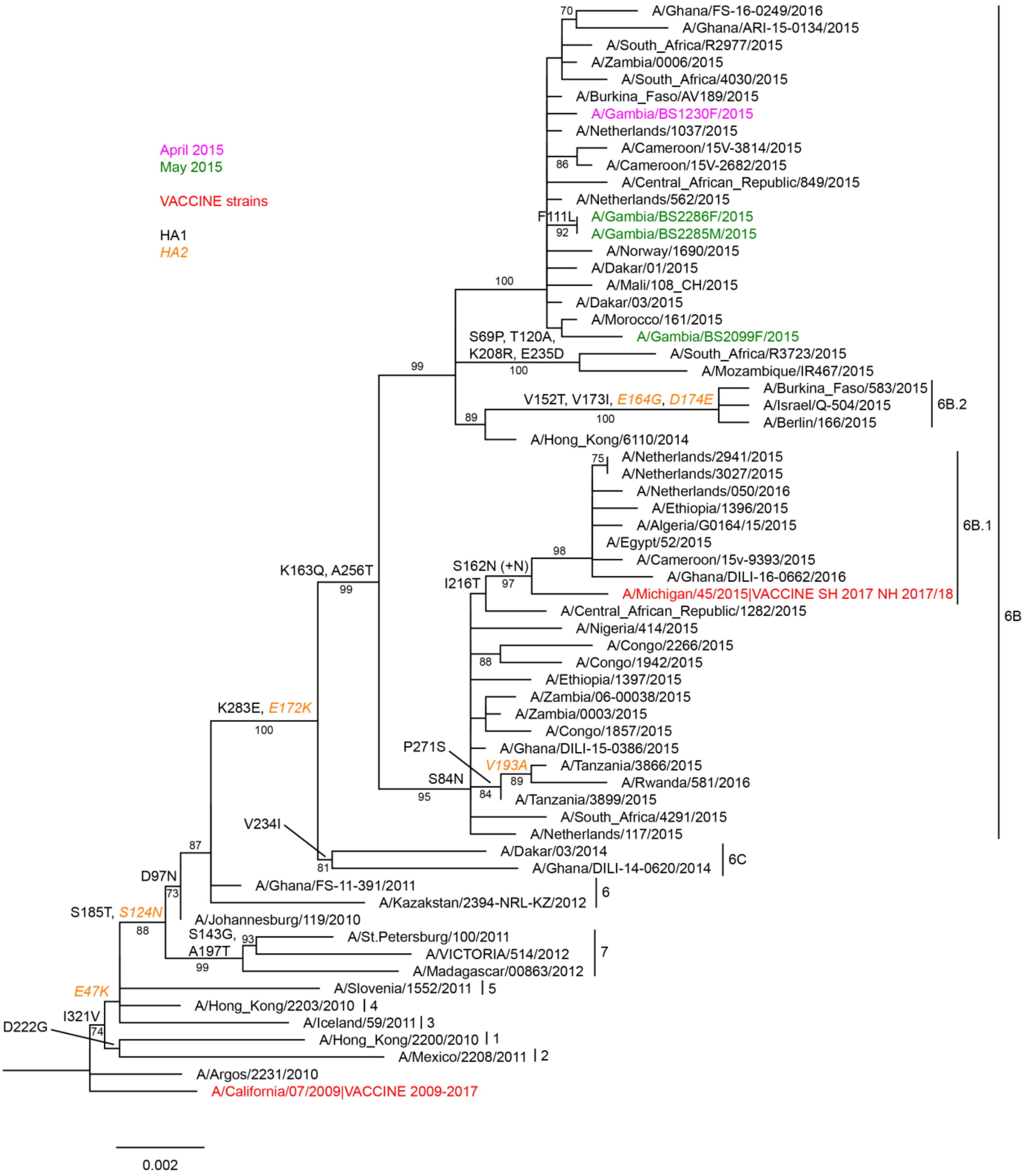

Note: strain A/Gambia/BS1230F/2015 is from a patient not resident in the BHDSS. Strains A/Gambia/BS2285M/2015 and A/Gambia/BS2286F/2015 are from the same patient; M = oropharyngeal swab; F = nasopharyngeal swab.

Figure S3. Phylogenetic tree for influenza virus-A(H1N1)pdm09, based on sequences of the full length hemagglutinin gene

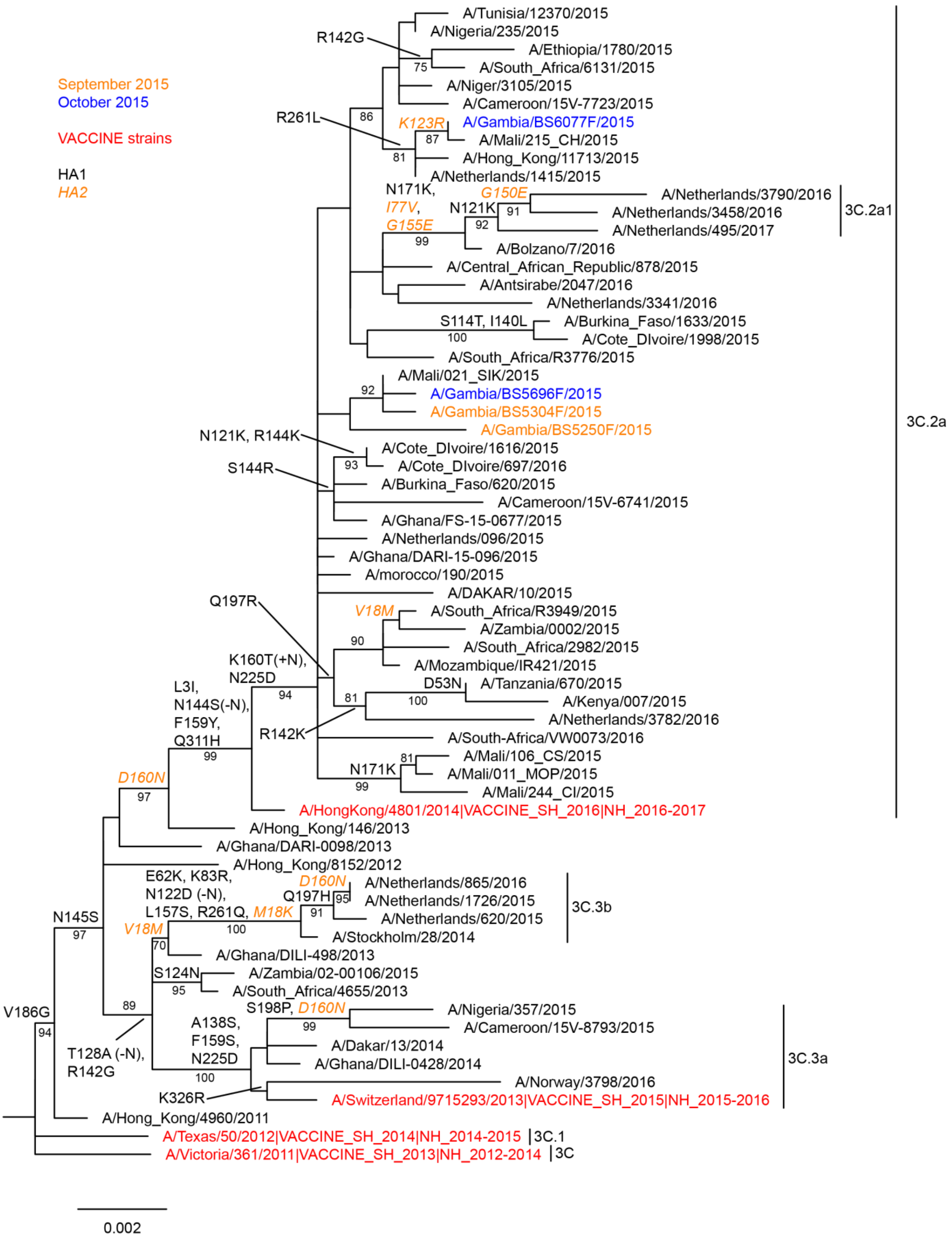

Figure S4. Phylogenetic tree for influenza virus-A(H3N2), based on sequences of the full length hemagglutinin gene

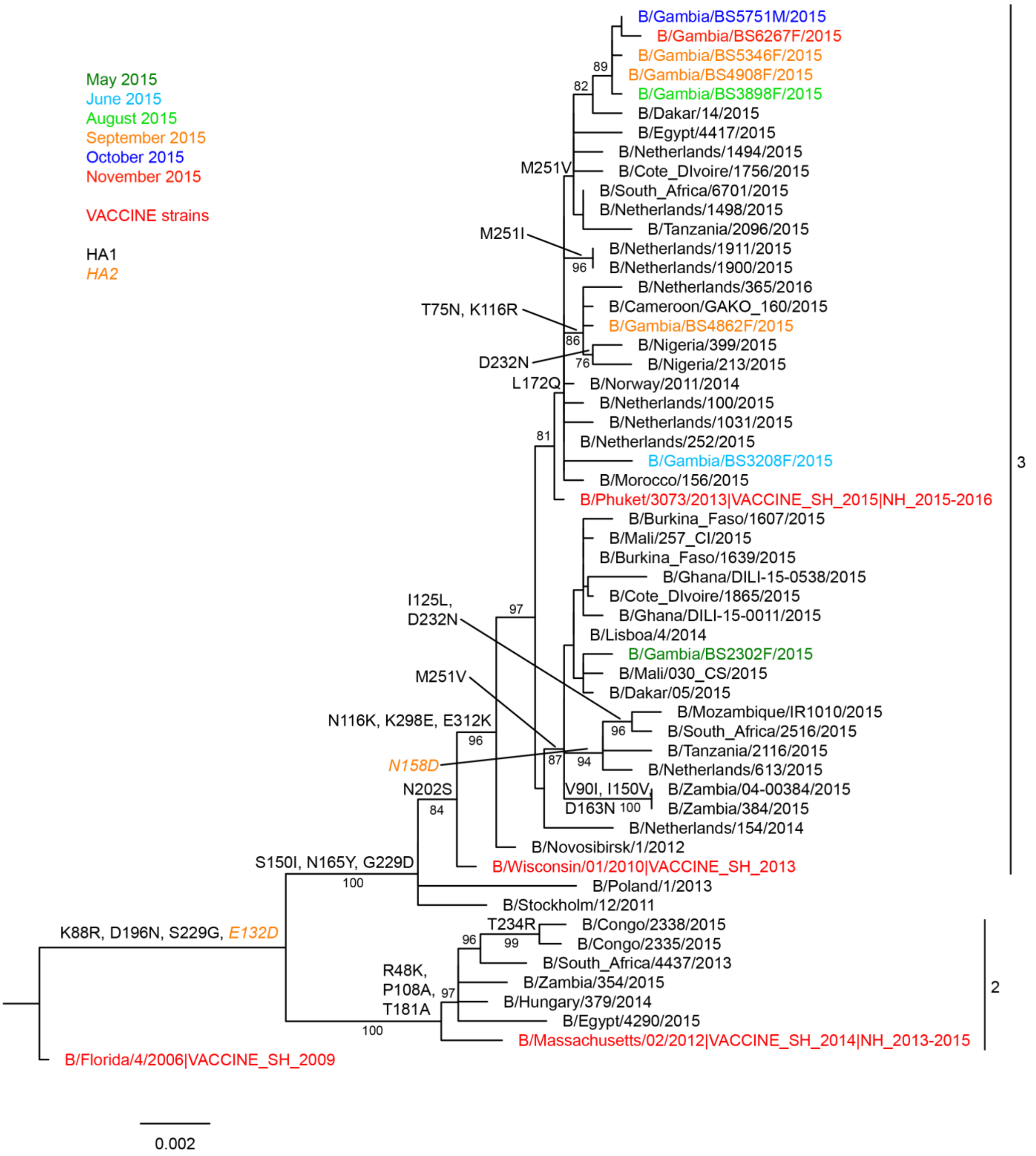

Figure S5. Phylogenetic tree for influenza virus-B/Yamagata lineage, based on sequences of the full length hemagglutinin gene

RSV F-protein amino acid composition analysis

The first 280 amino acids of the F-protein of 15 RSV-A and 2 RSV-B from The Gambia are shown in alignment with F-protein sequences of RSV-A strain A2 and RSV-B strain B-1 (GenBank accession numbers KJ155694 and AF013254 respectively), strains frequently used in vaccine development [1] in Figure S6. The corresponding Gambian partial RSV F protein gene sequences are available from GenBank under accession numbers MH399208-MH399224, MH686533 and MH686534. Potential N-glycosylation sites on the F-protein were predicted using the NetNGlyc server version 1.0.

Site Ø: prefusion F antigenic epitope targeted by many vaccines under development and monoclonal antibody MEDI8897 in phase II trial.[1,2,3] Site II: antigenic epitope targeted by palivizumab and motavizumab.[2] Site VIII: recently discovered prefusion F antigenic epitope.[3] Dot = the same amino acid as in the top sequence. X = amino acid unknown due to nucleotide ambiguity. - = not sequenced. Site Ø is conserved among RSV-A and RSV-B but of different composition between both types.[2] Site II is conserved between RSV-A and RSV-B strains, the reason why palivizumab and motavizumab act equally against both types.[2,3] Site VIII is conserved between RSV-A and RSV-B, although some differences exist that however do not impact reactivity of donor derived monoclonal antibodies with either type.[4] Orange shading indicates a potential N-glycosylation site.

1. Karron RA, Buchholz UJ, Collins PL. Live-attenuated respiratory syncytial virus vaccines. Curr Top Microbiol Immunol. 2013;372:259-84.
2. Meng J, Stobart CC, Hotard AL, Moore ML. An overview of respiratory syncytial virus. PLoS Pathog. 2014 Apr 24;10(4):e1004016.
3. Zhu Q, Lu B, McTamney P, Palaszynski S, Diallo S, Ren K, Ulbrandt ND, Kallewaard N, Wang W, Fernandes F, Wong S, Svabek C, Moldt B, Esser MT, Jing H, Suzich JA. Prevalence and significance of substitutions in the fusion protein of respiratory syncytial virus resulting in neutralization escape from antibody MEDI8897. J Infect Dis. 2018 Mar 30. doi: 10.1093/infdis/jiy189.
4. Mousa JJ, Kose N, Matta P, Gilchuk P, Crowe JE Jr. A novel pre-fusion conformation-specific neutralizing epitope on the respiratory syncytial virus fusion protein. Nat Microbiol. 2017 Jan 30;2:16271.

Figure S6. Composition of antigenic epitopes Ø, II and VIII in the F-protein sequences of RSV-A and RSV-B

```
                                          10        20        30        40        50        60        70
                                 ....|....|....|....|....|....|....|....|....|....|....|....|....|....|
                                                                                                Site Ø
HRSVA/strain A2/1961 KJ155694    MELLILKANAITTILTAVTFCFASGQNITEEFYQSTCSAVSKGYLSALRTGWYTSVITIELSNIKKNKCN
HRSVA/Gambia/BS0061F/2015        ------.T.......X..XL....S...X....................................E....
HRSVA/Gambia/BS0190F/2015        ...P...T.......A...L....S........................................E....
HRSVA/Gambia/BS0784F/2015        ...P...T.......A...L....S........................................E....
HRSVA/Gambia/BS2185F/2015        ...P...T.......A...L..T.S........................................E....
HRSVA/Gambia/BS2760F/2015        X..P...T.......A...L....S........................................E....
HRSVA/Gambia/BS3342F/2015        ...P...T.......A...L....S........................................E....
HRSVA/Gambia/BS3524F/2015        ~..P...T.......A...L....S........................................E....
HRSVA/Gambia/BS3882F/2015        ...P.H.T.......A...L....S........................................E....
HRSVA/Gambia/BS4142F/2015        ...P...T.......A...L....S........................................E....
HRSVA/Gambia/BS4525F/2015        ...P...T.......A...L.L..S........................................E....
HRSVA/Gambia/BS4844F/2015        ...P...T.......A...L....S........................................E....
HRSVA/Gambia/BS4862F/2015        ...P...T.......A...L....S........................................E....
HRSVA/Gambia/BS5232F/2015        ...P...T.......A...L....S........................................E....
HRSVA/Gambia/BS5346F/2015        ...P...T.......A...L....S........................................E....
HRSVA/Gambia/BS5921F/2015        ...P...T.......A...L....S........................................E....
HRSVB/strain B-1/1985 AF013254   .....HRLS..FLT.AINALYLT.S................R..F....................ET...
HRSVB/Gambia/BS1232F/2015        .....HRSS..FLT.AINALYLT.S................R.......................ET...
HRSVB/Gambia/BS2201F/2015        .....HRSS..FLT.AINALYLT.S................R.......................ET...
HRSVB/Gambia/BS5644F/2015        .....HRSS..FLT.AINALYLT.S................R.......................ET...
HRSVB/Gambia/BS5961F/2015        .....HRSS..FLT.AINALYLT.S................R.......................ET...
```

Alignment continued on next page

```
                                        80        90       100       110       120       130       140
                                ....|....|....|....|....|....|....|....|....|....|....|....|....|....|

HRSVA/strain A2/1961 KJ155694   GTDAKVKLIKQELDKYKNAVTELQLLMQSTPATNNRARRELPRFMNYTLNNAKKTNVTLSKKRKRRFLGF
HRSVA/Gambia/BS0061F/2015       ................................A.S................T.N................
HRSVA/Gambia/BS0190F/2015       ................................A.S................T.N................
HRSVA/Gambia/BS0784F/2015       ................................A.S................T.N................
HRSVA/Gambia/BS2185F/2015       ..............R.................A.S................T.N................
HRSVA/Gambia/BS2760F/2015       ................................A.S................T.N................
HRSVA/Gambia/BS3342F/2015       ................................A.S................T.N................
HRSVA/Gambia/BS3524F/2015       ................................A.S................T.N................
HRSVA/Gambia/BS3882F/2015       ................................A.S................T.N................
HRSVA/Gambia/BS4142F/2015       ................................A.S................T.N................
HRSVA/Gambia/BS4525F/2015       ................................A.S................T.N................
HRSVA/Gambia/BS4844F/2015       ................................A.S................T.N................
HRSVA/Gambia/BS4862F/2015       ................................A.S................T.N................
HRSVA/Gambia/BS5232F/2015       ................................A.S................T.N................
HRSVA/Gambia/BS5346F/2015       ................................A.S................T.N................
HRSVA/Gambia/BS5921F/2015       ................................A.S................T.N................
HRSVB/strain B-1/1985 AF013254  ...T........................N...A.......A.QY....I.TT.NL..SI...........
HRSVB/Gambia/BS1232F/2015       ...T........................N...V.......A.QY....I.TT.NL..LI...........
HRSVB/Gambia/BS2201F/2015       ...T........................N...V.......A.QY....I.TT.NL..LI......X....
HRSVB/Gambia/BS5644F/2015       ...T........................N...V.......A.QY....I.TT.NL..LI...........
HRSVB/Gambia/BS5961F/2015       ...T........................N...V.......A.QY....I.TT.NL..LI...........

                                       150       160       170       180       190       200       210
                                ....|....|....|....|....|....|....|....|....|....|....|....|....|....|
                                                         Site VIII                      Site Ø
HRSVA/strain A2/1961 KJ155694   LLGVGSAIASGVAVSKVLHLEGEVNKIKSALLSTNKAVVSLSNGVSVLTSKVLDLKNYIDKQLLPIVNKQ
HRSVA/Gambia/BS0061F/2015       ...........I..........................................................
HRSVA/Gambia/BS0190F/2015       ...........I..........................................................
HRSVA/Gambia/BS0784F/2015       ...........I..........................................................
HRSVA/Gambia/BS2185F/2015       ...........I..........................................................
HRSVA/Gambia/BS2760F/2015       ...........I..........................................................
HRSVA/Gambia/BS3342F/2015       ...........I..........................................................
HRSVA/Gambia/BS3524F/2015       ...........I..........................................................
HRSVA/Gambia/BS3882F/2015       ...........I..........................................................
HRSVA/Gambia/BS4142F/2015       ...........I..........................................................
HRSVA/Gambia/BS4525F/2015       ...........I..........................................................
HRSVA/Gambia/BS4844F/2015       ...........I..........................................................
HRSVA/Gambia/BS4862F/2015       ...........I..........................................................
HRSVA/Gambia/BS5232F/2015       ...........I..........................................................
HRSVA/Gambia/BS5346F/2015       ...........I..........................................................
HRSVA/Gambia/BS5921F/2015       ...........I..........................................................
HRSVB/strain B-1/1985 AF013254  ...........I................N..............................NN.......Q.
HRSVB/Gambia/BS1232F/2015       ...........I................N..QL..........................NN.......Q.
HRSVB/Gambia/BS2201F/2015       ...........I................N..QL..........................NN.......Q.
HRSVB/Gambia/BS5644F/2015       ...........I................N..QL..........................NN.......Q.
HRSVB/Gambia/BS5961F/2015       ...........I................N..QL..........................NN.......Q.

                                       220       230       240       250       260       270       280
                                ....|....|....|....|....|....|....|....|....|....|....|....|....|....|
                                                                                   Site II
HRSVA/strain A2/1961 KJ155694   SCSISNIETVIEFQQKNNRLLEITREFSVNAGVTTPVSTYMLTNSELLSLINDMPITNDQKKLMSNNVQI
HRSVA/Gambia/BS0061F/2015       .................................................................S....
HRSVA/Gambia/BS0190F/2015       .................................................................S....
HRSVA/Gambia/BS0784F/2015       .................................................................S....
HRSVA/Gambia/BS2185F/2015       .................................................................S....
HRSVA/Gambia/BS2760F/2015       .................................................................S....
HRSVA/Gambia/BS3342F/2015       .................................................................S....
HRSVA/Gambia/BS3524F/2015       .................................................................S....
HRSVA/Gambia/BS3882F/2015       .................................................................S....
HRSVA/Gambia/BS4142F/2015       .................................................................S....
HRSVA/Gambia/BS4525F/2015       .................................................................S....
HRSVA/Gambia/BS4844F/2015       .................................................................S....
HRSVA/Gambia/BS4862F/2015       .................................................................S....
HRSVA/Gambia/BS5232F/2015       .................................................................S....
HRSVA/Gambia/BS5346F/2015       .................................................................S....
HRSVA/Gambia/BS5921F/2015       .................................................................S....
HRSVB/strain B-1/1985 AF013254  ..R..............S.....N............L............................S....
HRSVB/Gambia/BS1232F/2015       ..R..............S..................L............................S....
HRSVB/Gambia/BS2201F/2015       ..R..............S..................L............................SX---
HRSVB/Gambia/BS5644F/2015       ..R..............S..................L............................S....
HRSVB/Gambia/BS5961F/2015       ..R..............S..................L............................S....
```

Note: strain HRSVA/Gambia/BS5921F/2015 is from a patient not resident in the BHDSS.

Results of RSV and influenza virus sequencing

Based on G-protein gene sequencing, all Gambian RSV-A viruses clustered in a recent clade designated ON1 characterized by a 72 nucleotides duplication insert.[1,2] All but one of these viruses clustered in a subgroup within this clade together with 2014 and 2015 viruses from The Netherlands and the USA in one group characterized by amino acid substitutions K134I, I243S (gain of O-glycosylation site) and E262K in the G-protein. Some further diversification was seen in these Gambian RSV-A with two groups of two viruses each, characterized by additional amino acid substitutions K197E and S294F (loss of O-glycosylation site) or L274P respectively. The one other Gambian RSV-A clustered within clade ON1 with 2015 and 2016 viruses from The Netherlands and New Zealand characterized by S102F (loss of O-glycosylation site), K216N and E271K. These results suggest multiple introductions and local expansion of RSV-A in The Gambia. Based on G-protein gene sequencing, all Gambian RSV-B viruses clustered in a recent clade previously designated BA and characterized by a 60 nucleotides duplication insert and a 6 nucleotide deletion.[1] The Gambian RSV-B viruses clustered with 2015 RSV-B from other continents in a subgroup of the BA clade characterized by amino acid substitutions T107A (loss of O-glycosylation site), R136T (gain of O-glycosylation site) and T303I. Additionally, one of the Gambian viruses lost two stop codons increasing the length of the protein. Similar to RSV-A, the results for Gambian RSV-B suggest a single introduction and local expansion. Although introduction followed by local expansion in a country has been shown before, e.g. for Kenya[3], a recent analysis of global RSV-A from all six continents strongly suggests intra- and inter-continent circulation.[2] Similar to described for Kenya[2,4], clustering of Gambian RSV-A and RSV-B with viruses from Europe suggests a transmission link between these continents, although Kenyan as well as Gambian RSV-A and RSV-B clustered also with viruses from other continents. In addition, the preliminary phylogenetic analysis with less complete Kenyan G-protein gene sequences showed that none of the Gambian RSV-A clustered with 2014 or 2015 Kenyan RSV-A. The vast majority of the Kenyan RSV-A segregated completely from the Gambian and other country RSV-A in a separate large group within clade ON1. In contrast, Gambian RSV-B clustered with a small proportion of 2015 and all 2016 Kenyan RSV-B in one bigger subgroup of the BA clade with RSV-B from other continents. These results suggest that there is no strong link between temporal circulation of RSV-A and RSV-B in West and East Africa. O- and N-glycosylation of the G-protein is important for antigenicity[5] and changes have been associated with repeat infection.[6] Clades RSV-A ON1 and RSV-B BA and subclades have been associated with specific glycosylation consensus patterns.[7] However, we showed that it is also important to analyze the effect of subgroup defining amino acid substitutions on the gain and loss of potential O- and N-glycosylation sites as that is the basis for the analysis of (sub)cluster-specific phenotypic properties of RSV.

Alignment of F-protein sequences showed that the antigenic sites Ø, II and VIII of Gambian RSV were highly conserved although some amino acid differences were detected in antigenic sites Ø and VIII between RSV-A and RSV-B. The Gambian F-proteins of RSV-A had the same potential N-glycosylation sites at amino acids 27, 70, 116, 120 and 126. The Gambian F-proteins of RSV-B had one site less at amino acid 126. Amino acid 70 is close to antigenic site Ø in the pre-fusion conformation and a loss of N-glycosylation at this site might affect the antigenicity of pre-F. Furthermore, the N-glycosylation pattern of the F-protein affects its fusion activity, of which the site at amino acid 500 which was not included in our analysis seems most important.[8] Nevertheless, N-glycosylation among the Gambian RSV was conserved for the other sites.

Influenza virus (IV) detected in The Gambia reflected the circulation of genotypes in the rest of Africa as well as in The Netherlands in 2015 with similar amino acid changes compared to vaccine strains recommended for the northern hemisphere (NH) 2014/15 season and the southern hemisphere (SH) 2015 season. Our results showed that the 2014/2015 NH and 2015 SH recommendations for A(H1N1)pdm09 vaccine strain were appropriate for A(H1N1)pdm09 viruses detected in The Gambia in 2015, as antigenic drift was minimal in A(H1N1)pdm09 viruses. For A(H3N2) there was a mismatch with recommendations for the 2014/2015 NH vaccine and the 2015 SH vaccine as all A(H3N2) detected in the Gambia belonged to clade 3C.2a, whereas the vaccine strains belonged to clades 3C.1 and 3C.3a respectively. For early circulation of B/Yamagata IV (all clade 3) in the Gambia, the 2014/2015 NH recommended vaccine strain was too far off (clade 2) whilst for late circulation of B/Yamagata the 2015 SH recommended vaccine strain was appropriate.

1. Bose ME, He J, Shrivastava S, Nelson MI, Bera J, Halpin RA, Town CD, Lorenzi HA, Noyola DE, Falcone V, Gerna G, De Beenhouwer H, Videla C, Kok T, Venter M, Williams JV, Henrickson KJ. Sequencing and analysis of globally obtained human respiratory syncytial virus A and B genomes. PLoS One. 2015 Mar 20;10(3):e0120098.
2. Otieno JR, Kamau EM, Oketch JW, Ngoi JM, Gichuki AM, Binter Š, Otieno GP, Ngama M, Agoti CN, Cane PA, Kellam P, Cotten M, Lemey P, Nokes DJ. Whole genome analysis of local Kenyan and global sequences unravels the epidemiological and molecular evolutionary dynamics of RSV genotype ON1 strains. Virus Evol. 2018 Sep 24;4(2):vey027.
3. Agoti CN, Otieno JR, Munywoki PK, Mwihuri AG, Cane PA, Nokes DJ, Kellam P, Cotten M. Local evolutionary patterns of human respiratory syncytial virus derived from whole-genome sequencing. J Virol. 2015 Apr;89(7):3444-54.
4. Elawar F, Griffiths CD, Zhu D, Bilawchuk LM, Jensen LD, Forss L, Tang J, Hazes B, Drews SJ, Marchant DJ. A Virological and Phylogenetic Analysis of the Emergence of New Clades of Respiratory Syncytial Virus. Sci Rep. 2017 Sep 25;7(1):12232.
5. Melero JA, Mas V, McLellan JS. Structural, antigenic and immunogenic features of respiratory syncytial virus glycoproteins relevant for vaccine development. Vaccine. 2017 Jan 11;35(3):461-468.
6. Okamoto M, Dapat CP, Sandagon AMD, Batangan-Nacion LP, Lirio IC, Tamaki R, Saito M, Saito-Obata M, Lupisan SP, Oshitani H. Molecular Characterization of Respiratory Syncytial Virus in Children With Repeated Infections With Subgroup B in the Philippines. J Infect Dis. 2018 Aug 24;218(7):1045-1053.
7. Schobel SA, Stucker KM, Moore ML, Anderson LJ, Larkin EK, Shankar J, Bera J, Puri V, Shilts MH, Rosas-Salazar C, Halpin RA, Fedorova N, Shrivastava S, Stockwell TB, Peebles RS, Hartert TV, Das SR. Respiratory Syncytial Virus whole-genome sequencing identifies convergent evolution of sequence duplication in the C-terminus of the G gene. Sci Rep. 2016 May 23;6:26311.
8. Zimmer G, Trotz I, Herrler G. N-glycans of F protein differentially affect fusion activity of human respiratory syncytial virus. J Virol. 2001 May;75(10):4744-51.
